# Supplementary material for: NODAL Secures Pluripotency upon Embryonic Stem Cell Progression from the Ground State
Source: Stem Cell Reports. 2017 Jun 29;9(1):77–91. doi: 10.1016/j.stemcr.2017.05.033 (PMC5511111; doi:10.1016/j.stemcr.2017.05.033)
Supplement: Document S1. Figures S1–S5 and Tables S1 and S2 [file mmc1.pdf]

Stem Cell Reports, Volume *9*

Supplemental Information

NODAL Secures Pluripotency upon Embryonic Stem Cell Progression from the Ground State

Carla Mulas, Tüzer Kalkan, and Austin Smith

Figure S1

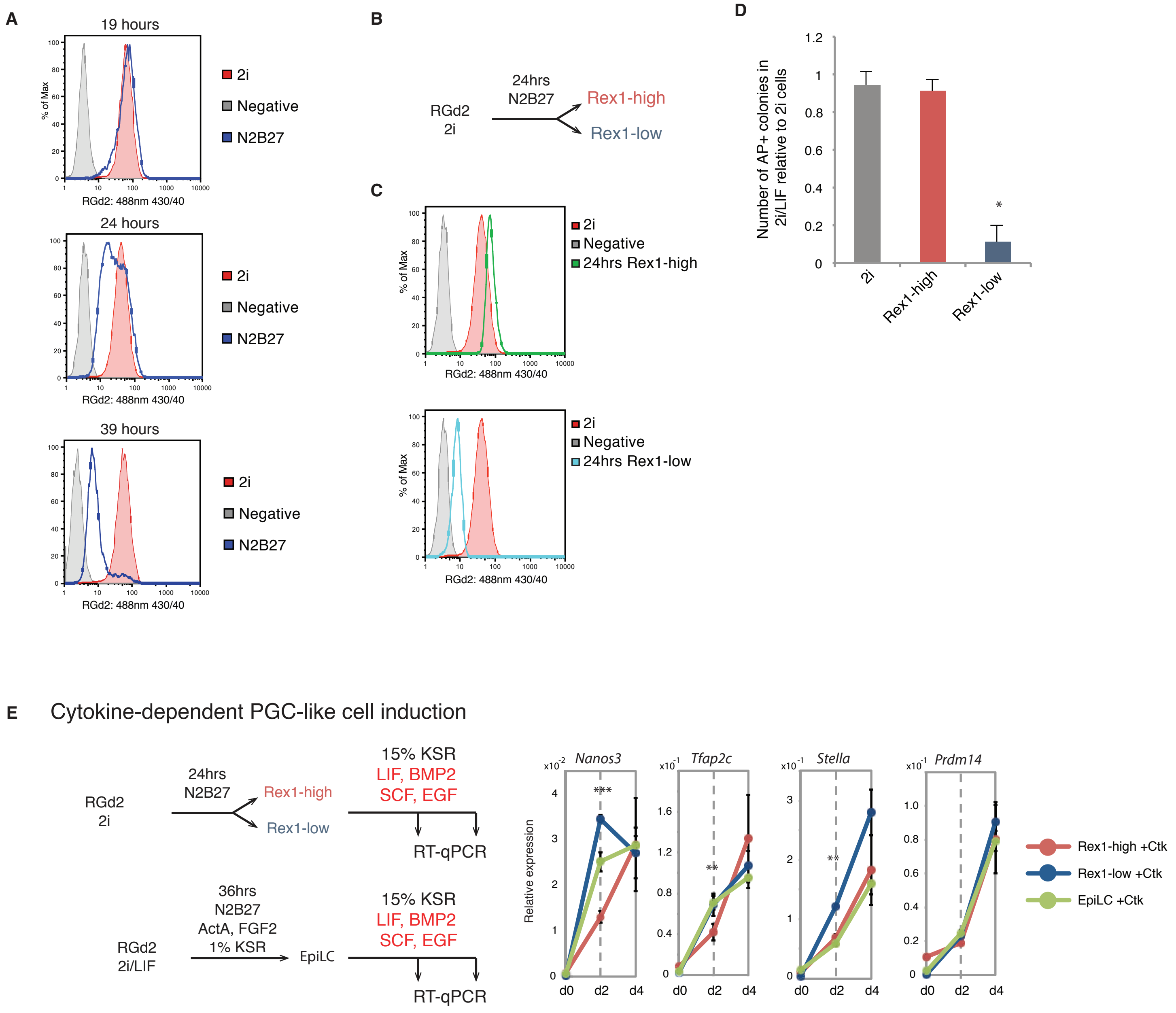
A
19 hours
24 hours
39 hours
% of Max
RGd2: 488nm 430/40
2i
Negative
N2B27
B
RGd2
2i
24hrs
N2B27
Rex1-high
Rex1-low
C
24hrs Rex1-high
24hrs Rex1-low
D
Number of AP+ colonies in 2i/LIF relative to 2i cells
2i
Rex1-high
Rex1-low
*
E
Cytokine-dependent PGC-like cell induction
15% KSR
LIF, BMP2
SCF, EGF
RT-qPCR
36hrs
N2B27
ActA, FGF2
1% KSR
RGd2
2i/LIF
EpiLC
Nanos3
Tfap2c
Stella
Prdm14
Relative expression
d0
d2
d4
***
**
Rex1-high +Ctk
Rex1-low +Ctk
EpiLC +Ctk

Figure S2

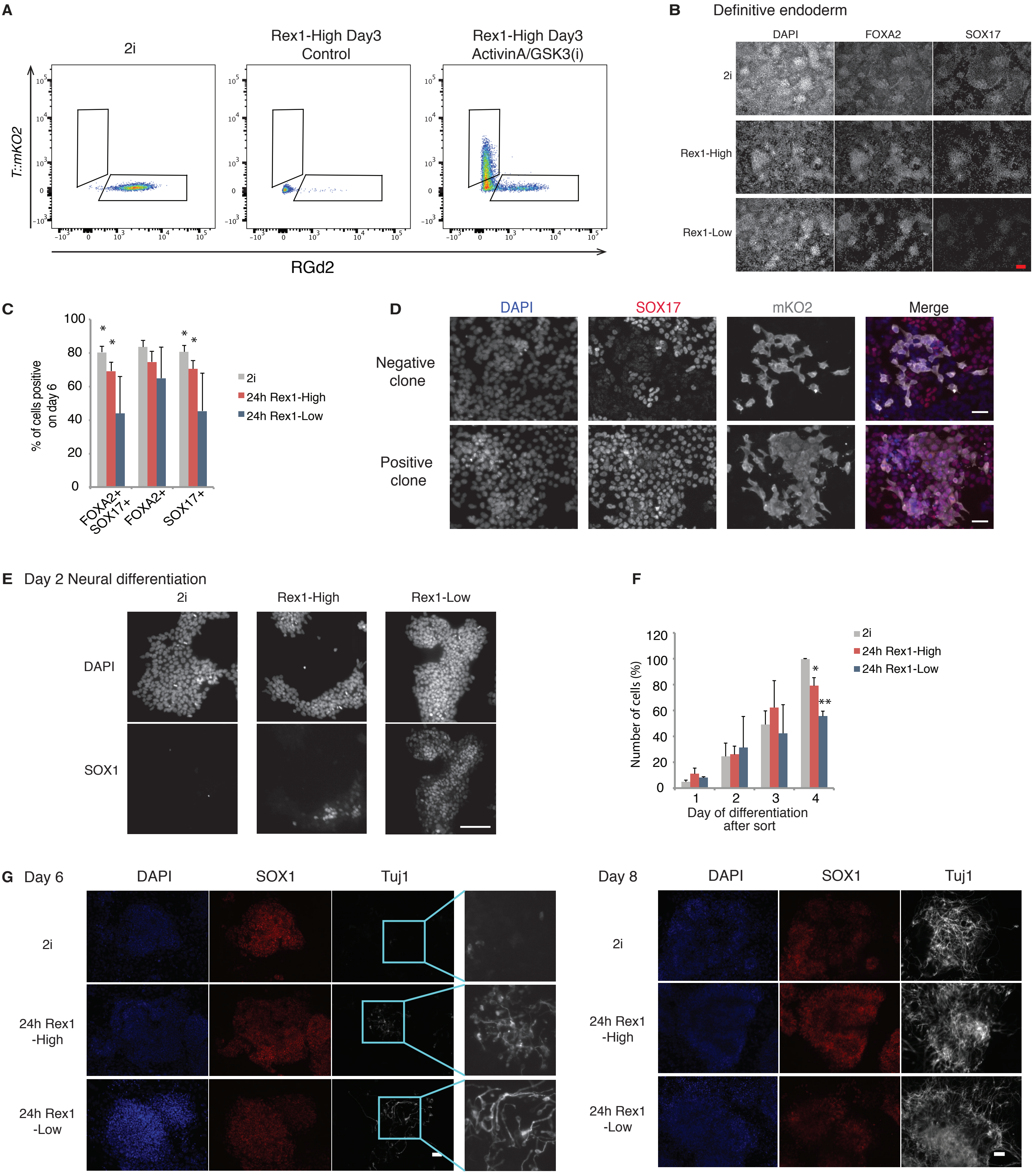
A
2i
Rex1-High Day3 Control
Rex1-High Day3 ActivinA/GSK3(i)
T::mKO2
RGd2
B
Definitive endoderm
DAPI
FOXA2
SOX17
2i
Rex1-High
Rex1-Low
C
% of cells positive on day 6
FOXA2+ SOX17+
FOXA2+
SOX17+
2i
24h Rex1-High
24h Rex1-Low
D
DAPI
SOX17
mKO2
Merge
Negative clone
Positive clone
E
Day 2 Neural differentiation
2i
Rex1-High
Rex1-Low
DAPI
SOX1
F
Number of cells (%)
Day of differentiation after sort
2i
24h Rex1-High
24h Rex1-Low
G
Day 6
DAPI
SOX1
Tuj1
2i
24h Rex1 -High
24h Rex1 -Low
Day 8
DAPI
SOX1
Tuj1
2i
24h Rex1 -High
24h Rex1 -Low

Figure S3

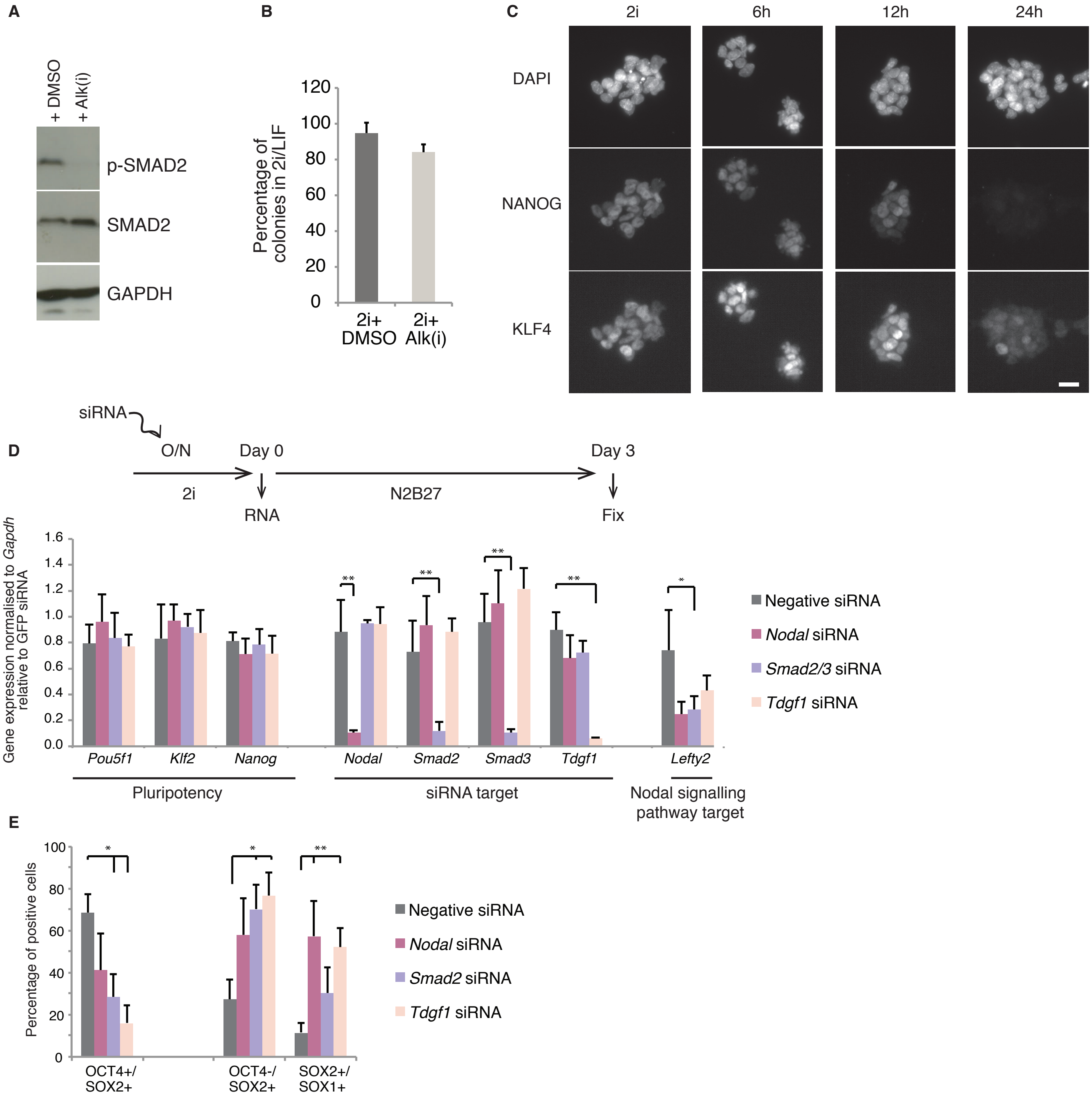

A
+ DMSO
+ Alk(i)
p-SMAD2
SMAD2
GAPDH
B
Percentage of colonies in 2i/LIF
120
100
80
60
40
20
0
2i+ DMSO
2i+ Alk(i)
C
2i
6h
12h
24h
DAPI
NANOG
KLF4
D
siRNA
O/N
Day 0
Day 3
2i
N2B27
RNA
Fix
Gene expression normalised to Gapdh relative to GFP siRNA
1.6
1.4
1.2
1.0
0.8
0.6
0.4
0.2
0.0
Pou5f1
Klf2
Nanog
Nodal
Smad2
Smad3
Tdgf1
Lefty2
Pluripotency
siRNA target
Nodal signalling pathway target
Negative siRNA
Nodal siRNA
Smad2/3 siRNA
Tdgf1 siRNA
E
Percentage of positive cells
100
80
60
40
20
0
OCT4+/ SOX2+
OCT4-/ SOX2+
SOX2+/ SOX1+
Negative siRNA
Nodal siRNA
Smad2 siRNA
Tdgf1 siRNA

Figure S4

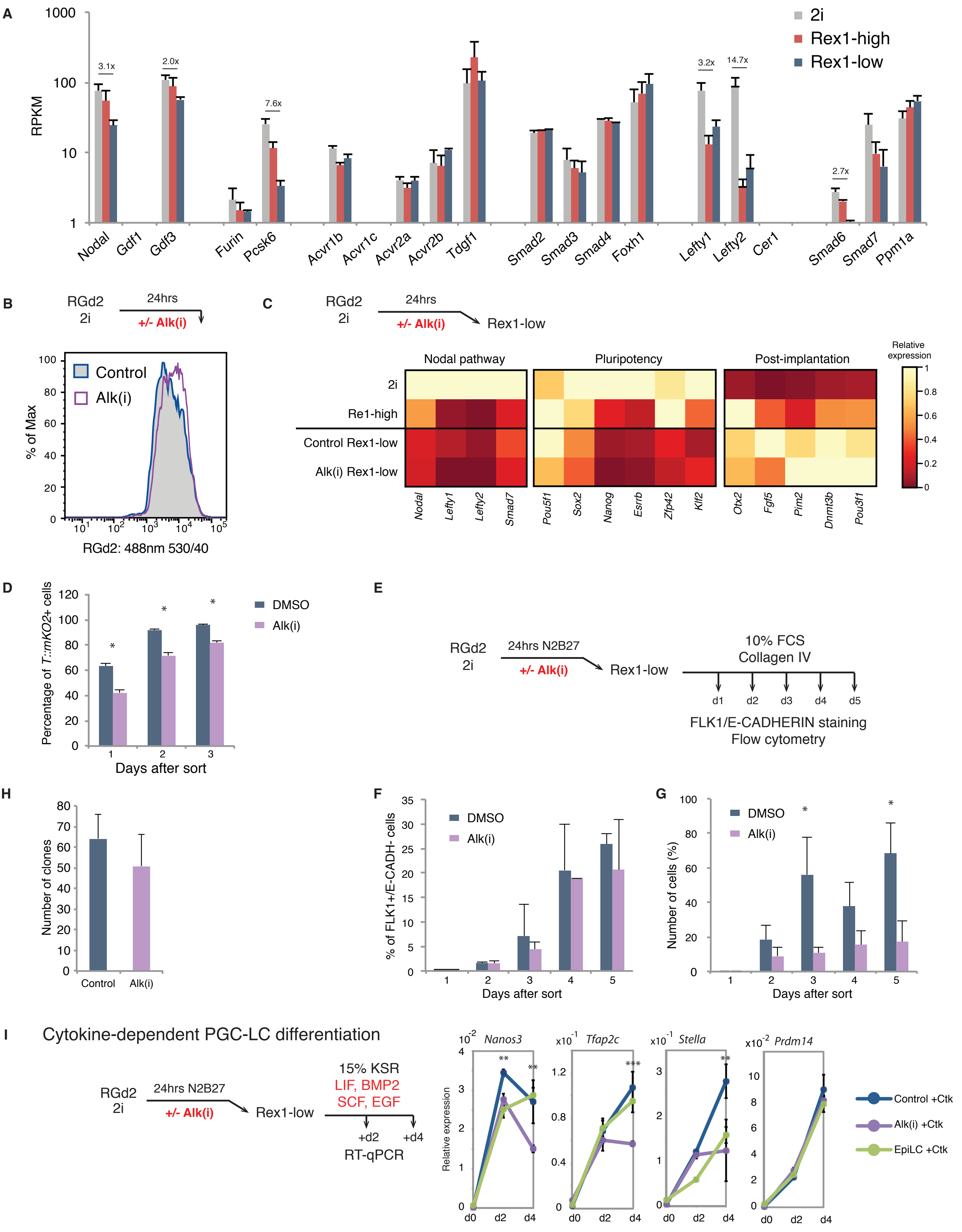

A
RPKM
1000
100
10
1
3.1x
2.0x
7.6x
3.2x
14.7x
2.7x
2i
Rex1-high
Rex1-low
Nodal
Gdf1
Gdf3
Furin
Pcsk6
Acvr1b
Acvr1c
Acvr2a
Acvr2b
Tdgf1
Smad2
Smad3
Smad4
Foxh1
Lefty1
Lefty2
Cer1
Smad6
Smad7
Ppm1a
B
RGd2
2i
24hrs
+/- Alk(i)
Control
Alk(i)
% of Max
RGd2: 488nm 530/40
C
RGd2
2i
24hrs
+/- Alk(i)
Rex1-low
Nodal pathway
Pluripotency
Post-implantation
Relative expression
2i
Re1-high
Control Rex1-low
Alk(i) Rex1-low
Nodal
Lefty1
Lefty2
Smad7
Pou5f1
Sox2
Nanog
Esrrb
Zfp42
Klf2
Otx2
Fgf5
Pim2
Dnmt3b
Pou3f1
D
Percentage of T::mKO2+ cells
DMSO
Alk(i)
Days after sort
E
RGd2
2i
24hrs N2B27
+/- Alk(i)
Rex1-low
10% FCS
Collagen IV
d1
d2
d3
d4
d5
FLK1/E-CADHERIN staining
Flow cytometry
H
Number of clones
Control
Alk(i)
F
% of FLK1+/E-CADH- cells
DMSO
Alk(i)
Days after sort
G
Number of cells (%)
DMSO
Alk(i)
Days after sort
I
Cytokine-dependent PGC-LC differentiation
RGd2
2i
24hrs N2B27
+/- Alk(i)
Rex1-low
15% KSR
LIF, BMP2
SCF, EGF
+d2
+d4
RT-qPCR
Relative expression
Nanos3
Tfap2c
Stella
Prdm14
d0
d2
d4
Control +Ctk
Alk(i) +Ctk
EpiLC +Ctk

Figure S5

A *Nodal* locus

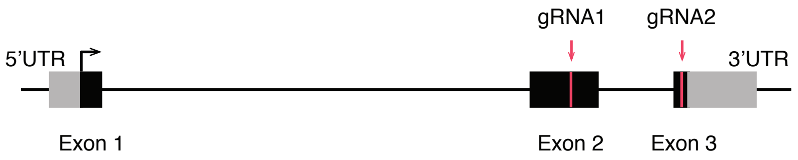

B

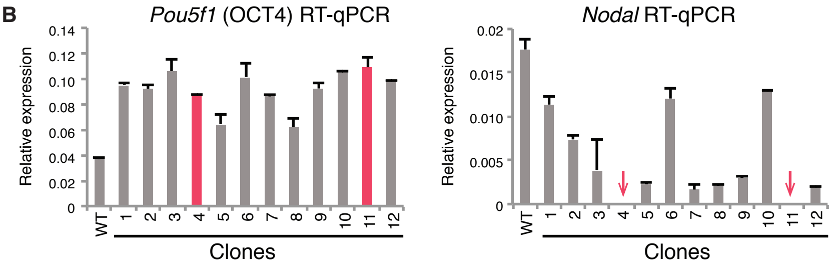

Table S1 – Antibodies used in this study

Antigen	Supplier	Cat. No	Dilution
SOX1	Cell Signalling	4194	1:200
OCT4	Santa Cruz	sc-5279 or sc-8628	1:400
NANOG	eBioscience	14-5761-80	1:200
KLF4	Abcam	ab72543	1:300
Tuj1	R&D	MAB1195	1:500
FOXA2	Abcam	ab40874	1:200
SOX17	R&D	AF1924	1:200
BRACHYURY	R&D	AF2085	1:200
ESRRB	Perseus	PP-H6705-00	1:300
mKO2	Amalgaam-MBL	M168-3	1:1000
BLIMP1	eBiosciences	14-5963-82	1:50
SMAD2	Cell Signalling	3101	1:1000
p-SMAD2	Cell Signalling	3103	1:1000
GAPDH	Sigma-Aldrich	G8795	1:5000
E-CADHERIN	eBioscience	50-3249-82	
CXCR4	BD Biosciences	552967 or 558644	
FLK1	BD Biosciences	562941	

Table S2 – Primers and probes used in this study

Target	Forward primer	Reverse primer	UPL probe
Actb	ggagattactgctctggctcc	acagagtacttgcgctcaggagg	-
Gdf3	tgttcgtgggaacctgct	ccatcttggaaaggtttctgtg	-
Lefty2	cacaagttggtccgtttcg	ggtacctcggggtcacaat	78
Nanos3	caaggcaaagacacaggatg	cttcctgccacttttggaac	25
Nodal	ccaaccatgcctacatcca	cacagcacgtggaaggaac	40
Pou3f1	catttttcgtttcgttttaccc	gagcgcagaccctctctg	72
Pou3f3	tctgagaccgcccacaag	gagcggcagtcagcaaag	22
Prdm1 UTR	ggtgaatcagggtgccttta	gagaggtgcagggaagcac	109
Prdm14 UTR	aaaatgaccttgaattacaggattaag	cataccaaatctctaggtagtgcaaa	76
Smad2	taagaatgagttttgtgaagggc	agcaaggagtacttgttactgtctg	-
Smad3	tccgtatgagcttcgtcaaagg	tagctcaatccagcagggg	-
Stella	tggaaattagaacgtacatactccaa	gatgcacaacgatccagattt	73
Tdgf1	gtttgaatttggacccgttg	ggaaggcacaaactggaaag	93
Tfap2c UTR	aaaagaggaaggaaacaggaaag	gggtcccctgtttttaagga	40
Zic1	aacctcaagatccacaaaagga	cctcgaactcgcacttgaa	7

TaqMan probes

Pou5f1
Sox2
Nanog
Esrrb
Zfp42
Klf2
Otx2
Fgf5
Pim2
Sox1
Dnmt3b
Gapdh

SUPPLEMENTAL FIGURE LEGENDS

Figure S1 – related to Figure 1

(**A**) Flow cytometry histogram of RGd2 cells after removal of 2i.

(**B**) Experimental set up for sorting experiments.

(**C**) Flow cytometry profile of sorted fractions.

(**D**) Replating capacity of 2i, 24hrs Rex1-high and 24hrs Rex1-low cells in 2i/LIF media.

(**E**) Experimental set up for cytokine-dependent PGCLC induction and RT-qPCR of endogenous PGC-associated transcripts. *** $p<0.0001$, **$p<0.001$ in pairwise comparison of Rex1-high and Rex1-low.

Figure S2 – related to Figure 2

(**A**) Flow cytometry plots of RGd2+*T::mKO2* cells in 2i, and Rex1-high sorted cells for 3 days in control or ActivinA+GSK3(i).

(**B**) Representative images of definitive endoderm differentiation immunostained for FOXA2 and SOX17. Scale bar= 50μm.

(**C**) Quantification of the percentage of cells positive for SOX17 and FOXA2 during definitive endoderm differentiation.

(**D**) Representative images of clonal definitive endoderm differentiation, showing an example of a clone predominantly SOX17 negative and a SOX17 positive clone.

(**E**) Representative images of neural differentiation on day 2. Scale bar= 50μm.

(**F**) Normalised number of cells during neural differentiation.

(**G**) Immunostaining for SOX1 and Tuj1 of 2i, Rex1-high and Rex1-low cells after 6 and 8 days of differentiation. Scale bar= 50μm.

To determine the normalised number of cells as a percentage for each biological replicate, the number of cells was normalised by the highest value obtained in that biological replicate.

*$p<0.01$

Figure S3 – related to Figure 3

(**A**) Western blot showing p-SMAD2, SMAD2 and GADPH protein in cells treated with control or Alk(i) for 30min.

(**B**) Number of colonies of ES cells grown in 2i+DMSO or 2i+Alk(i) for three passages.

(**C**) Representative images of NANOG and KLF4 immunostaining during the first 24hrs of differentiation (intensities have been enhanced for visualisation purposes). Scale bar= 20 μm.

(**D**) RT-qPCR of *Nodal*, *Tdgf1* and *Smad2/3* siRNA treated cells after overnight transfection in 2i. siRNA knockdown did not affect the expression of pluripotency genes *Pou5f1, Klf4* or *Nanog* but in some cases it did affect the expression of the NODAL signalling target *Lefty2.*

(**E**) Quantification of the number of OCT4/SOX2 double positive cells, OCT4 negative/SOX2 positive and SOX2/SOX1 double positive cells on day 3 of neural differentiation after treatment with siRNA.

* p <0.05, ** p<0.01

Figure S4 – related to Figures 4 and 5

(**A**) Expression of NODAL pathway signalling components in 2i, Rex1-high and Rex1-low cells (Kalkan et al. 2017). Fold changes are shown for genes showing a significant difference between 2i and Rex1-low cells (p<0.05).

(**B**) NODAL inhibition before and during downregulation of Rex1 – Flow cytometry plot of RGd2 cells differentiated for 24hrs in Alk(i) or control (DMSO).

(**C**) Relative expression of NODAL target genes, pluripotency and differentiation factors in Rex1-low cells arising from control or Alk(i) conditions by RT-qPCR. 2i and Rex1-high cells are included as controls.

(**D**) Percentage of *T::mKO2* positive cells during ActivinA/GSK3(i) treatment of control or Alk(i) derived Rex1-low cells.

(**E**) Lateral mesoderm differentiation of 24hrs Alk(i) or control treated Rex1-low cells.

(**F**) Percentage of FLK1+/E-CADH- cells.

(**G**) Number of clones after 4 days of definitive endoderm differentiation.

To determine the normalised number of cells as a percentage for each biological replicate, the number of cells was normalised by the highest value obtained in that biological replicate.

(**H**) Histogram showing the normalised number of cells.

(**I**) Experimental set up for cytokine-dependent PGCLC induction and RT-qPCR of endogenous PGC-associated transcripts of control or Alk(i)-treated cells.

*p<0.05, **p<0.001, *** p<0.0001 in pairwise comparison of Rex1-high and Rex1-low.

Figure S5 – related to figure 6

(**A**) Strategy for inactivating *Nodal*

(**B**) RT-qPCR for *Pou5f1* (Oct4) and *Nodal* in picked clones. Clones 4 and 11 were negative for *Nodal* mRNA and were used for subsequent analysis
