# Supplementary material for: NODAL Secures Pluripotency upon Embryonic Stem Cell Progression from the Ground State
Source: Stem Cell Reports. 2017 Jun 29;9(1):77–91. doi: 10.1016/j.stemcr.2017.05.033 (PMC5511111; doi:10.1016/j.stemcr.2017.05.033)
Supplement: Document S2. Article plus Supplemental Information [file mmc2.pdf]

Stem Cell Reports
Article

ISSCR

OPEN ACCESS

NODAL Secures Pluripotency upon Embryonic Stem Cell Progression from the Ground State

Carla Mulas,[1,2] Tüzer Kalkan,[1] and Austin Smith[1,2,*]
[1]Wellcome Trust – Medical Research Council Stem Cell Institute, University of Cambridge, Tennis Court Road, Cambridge CB2 1QR, UK
[2]Department of Biochemistry, University of Cambridge, Tennis Court Road, Cambridge CB2 1GA, UK
*Correspondence: austin.smith@cscr.cam.ac.uk
http://dx.doi.org/10.1016/j.stemcr.2017.05.033

SUMMARY

Naive mouse embryonic stem cells (ESCs) can develop multiple fates, but the cellular and molecular processes that enable lineage competence are poorly characterized. Here, we investigated progression from the ESC ground state in defined culture. We utilized downregulation of *Rex1*::*GFPd2* to track the loss of ESC identity. We found that cells that have newly downregulated this reporter have acquired capacity for germline induction. They can also be efficiently specified for different somatic lineages, responding more rapidly than naive cells to inductive cues. Inhibition of autocrine NODAL signaling did not alter kinetics of exit from the ESC state but compromised both germline and somatic lineage specification. Transient inhibition prior to loss of ESC identity was sufficient for this effect. Genetic ablation of *Nodal* reduced viability during early differentiation, consistent with defective lineage specification. These results suggest that NODAL promotes acquisition of multi-lineage competence in cells departing naive pluripotency.

INTRODUCTION

Pluripotency denotes a flexible cellular potential to differentiate into all lineages of the developing embryo. In mammals this property emerges in the epiblast of the pre-implantation blastocyst (Boroviak et al., 2014; Gardner, 1975; Rossant, 1975). After implantation, epiblast cells remain pluripotent while undergoing profound cellular and molecular changes in preparation for gastrulation (Smith, 2017). In mice the post-implantation epiblast develops into a cup-shaped epithelium, the egg cylinder. Signaling cues from extra-embryonic tissues then pattern the egg cylinder to establish anterior-posterior and proximal-distal axes prior to lineage specification (reviewed in Arnold and Robertson, 2009; Rossant and Tam, 2009).

In mouse the naive phase of pluripotency can be captured in culture in the form of embryonic stem cells (ESCs) (reviewed by Nichols and Smith, 2012). Dual inhibition (2i) of MEK1/2 and glycogen synthase kinase 3 (GSK3) (Ying et al., 2008), optionally in combination with the cytokine leukemia inhibitory factor (LIF), allows mouse ESCs to maintain the transcription profile, DNA hypomethylation status, and developmental potential characteristic of the pre-implantation epiblast from which they are derived (Boroviak et al., 2014, 2015; Habibi et al., 2013; Leitch et al., 2013). ESCs in 2i are stable and relatively homogeneous, a condition referred to as "ground state" (Marks et al., 2012; Wray et al., 2010). Such uniformity in defined conditions provides an experimental system to characterize cellular and molecular events that generate multiple lineage-committed states from a developmental blank canvas.

ESC progression from the ground state is initiated simply by removal of the inhibitors. In adherent culture this results predominantly in neural specification (Ying et al., 2003) or in a mixture of neural and mesoendodermal fates, depending on cell density (Kalkan et al., 2017). Previous studies have identified expression of REX1 (gene name *Zfp42*) as a marker of undifferentiated ESCs that is downregulated prior to lineage specification (Betschinger et al., 2013; Kalkan et al., 2017; Kalkan and Smith, 2014; Leeb et al., 2014; Toyooka et al., 2008; Wray et al., 2010, 2011; Yang et al., 2012). In this study, we exploit a *Rex1*::*GFPd2* (RGd2) reporter cell line (Kalkan et al., 2017) to isolate cells at initial stages of progression from naive pluripotency following release from 2i in adherent serum-free culture. We examine whether cells exiting the ESC state guided by autocrine cues commit preferentially to a neural fate or exhibit competence for multi-lineage differentiation.

RESULTS

Multi-lineage Differentiation Capacity Is Manifest after Loss of Naive ESC Identity

In *Rex1*::*GFPd2* (RGd2) reporter ESCs, a short-half-life GFP is expressed from the endogenous REX1 (*Zfp42*) locus (Marks et al., 2012; Wray et al., 2011). Loss of the reporter coincides with downregulation of naive pluripotency factors and functionally with extinction of clonal self-renewal capacity (Kalkan et al., 2017) (Figures S1A–S1D). GFP downregulation is asynchronous across the population. For at least 16 hr cells remain uniformly GFP high, but by 24 hr expression is heterogeneous and in a minority of cells the reporter has been downregulated (Kalkan et al., 2017).

CrossMark

This is an open access article under the CC BY license (http://creativecommons.org/licenses/by/4.0/).

Rex1-low cells have lost the capacity to resume self-renewal in 2i/LIF, whereas cells with high GFP produce undifferentiated ESC colonies with the same efficiency as cells in the initial 2i culture (Figure S1D, see also Kalkan et al., 2017). We focused attention on the character of cells 24 hr after 2i withdrawal, the first time point at which it is practical to isolate a substantial population of Rex1-low cells by flow cytometry (Kalkan et al., 2017).

We first investigated capacity to form primordial germ cell (PGC)-like cells (PGCLCs). Previous studies have shown that undifferentiated ESCs are not directly competent for germline specification but must first transition to a transient epiblast-like (EpiLC) population which can then be induced to form PGCLCs (Hayashi et al., 2011; Nakaki et al., 2013). The EpiLC population is obtained by transfer from 2i/LIF to N2B27 medium supplemented with activin A, basic fibroblast growth factor (FGF), and the serum substitute KSR (Knockout Serum Replacement) for 48 hr (Hayashi et al., 2011). We assessed whether the first cells that exit the ground state in N2B27 alone exhibit competence to form PGCLCs. For this purpose we used RGd2 ESCs transfected with a doxycycline (Dox)-regulatable expression construct containing the three germline determination factors *Prdm1* (BLIMP1), *Prdm14*, and *Tfap2c* (Magnúsdóttir et al., 2012; Nakaki et al., 2013). Stable transfectants were withdrawn from 2i for 24 hr and the high and low GFP fractions isolated by fluorescence-activated cell sorting (FACS) (Figure 1A). Sorted cells (2,500) were aggregated with or without Dox in non-adherent 96-well plates in medium containing 15% KSR (Nakaki et al., 2013). After 4 days, the expression of OCT4 and BLIMP1 protein was analyzed. Dual expression of BLIMP1 and OCT4 is a combination unique to PGCs and PGCLCs (Hayashi et al., 2011; Kurimoto et al., 2008; Nakaki et al., 2013). Furthermore, undifferentiated ESCs do not tolerate appreciable levels of BLIMP1 protein (Magnúsdóttir et al., 2013). In the absence of Dox, few cells co-expressing BLIMP1 with OCT4 were present in aggregates from either population (Figure 1B). Dox treatment induced double-positive cells from the Rex1-low fraction but had little effect on the Rex1-high cells (Figures 1B and 1C). Quantitative imaging analysis confirmed a higher number of cells were double-positive for OCT4 and BLIMP1 in cultures derived from Rex1-low cells (Figure 1D), at a frequency comparable with that previously reported for EpiLCs (Nakaki et al., 2013). By qRT-PCR analysis we detected upregulated expression of endogenous *Prdm1* (BLIMP1), along with *Prdm14*, *Tfap2c*, *Nanos3*, and *Stella*, as well as maintenance of *Pou5f1* (OCT4) (Figure 1E). *T* (BRACHYURY) was induced transiently on day 2 as previously described for PGCLC induction (Figure 1E) (Nakaki et al., 2013). We also carried out cytokine induction of PGCLCs and observed earlier upregulation of PGC markers *Nanos3*, *Tfap2c*, and *Stella* in Rex1-low cells compared with Rex1-high cells (Figure S1E). The kinetics of upregulation and overall expression levels of PGC markers were comparable with those for EpiLC treated in parallel (Figure S1E). Thus, ESCs newly exited from the ground state under autocrine stimulation in defined conditions have acquired competence for germline specification.

We then examined somatic lineage potential of Rex1-low cells. Sorted fractions were plated in media that favor mesoderm, definitive endoderm, or neural lineages, respectively, and the timing and efficiency of differentiation quantified.

Activin A combined with GSK3 inhibition (GSK3(i)) elicits the upregulation of primitive-streak markers such as BRACHYURY (*T*) in differentiating ESCs (Gadue et al., 2006; Morrison et al., 2015; Tsakiridis et al., 2014; Turner et al., 2014). We modified RGd2 cells to express an mKO2 fluorescent reporter from the *T* locus (Figure 2A). *T*::*mKO2* was not expressed in undifferentiated ESCs in 2i (Figure S2A), and not detected until day 3 of treatment with activin A plus GSK3(i). In contrast, Rex1-low cells replated in the presence of activin A and GSK3(i) upregulated *T*::*mKO2* after 1 day and all cells were positive by day 2. Rex1-high cells upregulated *T*::*mKO2* more slowly and some cells remained GFP high even after 3 days, indicating they remained undifferentiated and unresponsive to differentiation cues (Figure 2B).

To test further differentiation, we plated the sorted fractions in conditions that promote lateral mesoderm (Nishikawa et al., 1998; Yamashita et al., 2000). All populations gave rise to FLK1-positive/E-CADHERIN-negative cells after 4–5 days (Figure 2C).

We assessed definitive endoderm differentiation after sorting by measuring the percentage of CXCR4/E-CADHERIN double-positive cells (Morrison et al., 2008; Yasunaga et al., 2005) under specific inductive conditions (Morrison et al., 2015) (Figure 2D). Compared with 2i cells or the Rex1-high population, fewer double-positive cells accumulated from the Rex1-low cells (Figure 2E). However, we noted that the majority of Rex1-low cells died after replating in these conditions (Figure 2F). The survivors formed SOX17/FOXA2 double-positive cells, and every SOX17-positive cell was positive for FOXA2, substantiating endoderm identity (Burtscher and Lickert, 2009) (Figures S2B and S2C). Induction of the later marker, SOX17, was reduced from Rex1-low cells compared with Rex1-high or 2i cells. We hypothesized that sorted Rex1-low cells might have impaired survival and differentiation because of a requirement for high cell density in the endoderm program. We therefore combined sorted cells with unsorted populations to reproduce the density of non-manipulated cultures (Figure 2G). To trace the sorted cell progeny, we employed RGd2 cells constitutively labeled with mKO2 under the control of a CAG promoter (Niwa et al., 1991). Two hundred

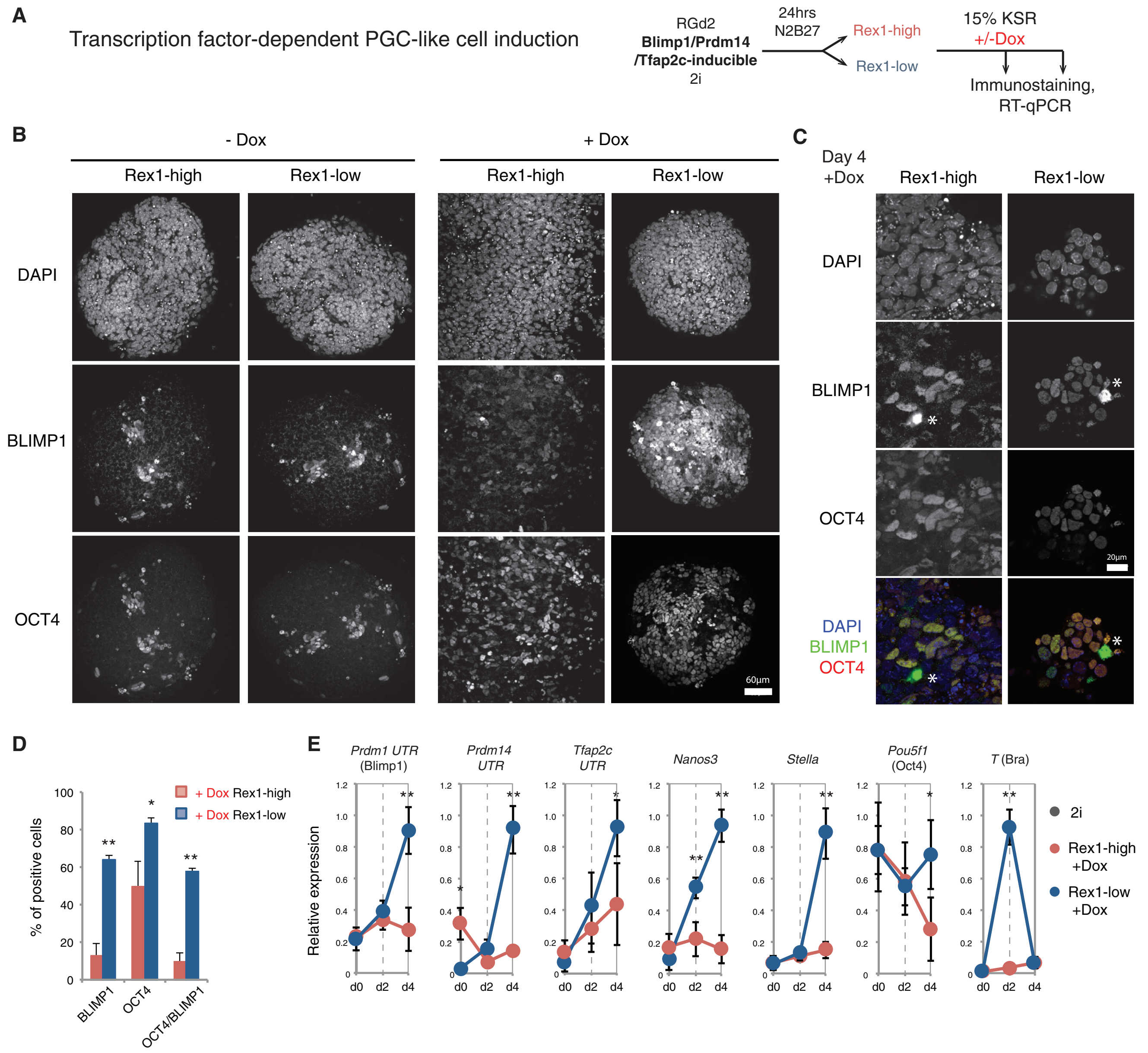

Figure 1. Acquisition of PGCLC Differentiation Capacity

(A) Experimental setup for transcription factor-dependent PGCLC specification.

(B) Expression of BLIMP1 and OCT4 in day-4 aggregates differentiated in the presence or absence of Dox to induce transcription factor overexpression. Scale bar, 60 μm.

(C) Zoom-in of the expression of BLIMP1 and OCT4 in day-4 aggregates differentiated in the presence or absence of Dox to induce transcription factor overexpression. Asterisks indicate overexpression staining artifacts. Scale bar, 20 μm.

(D) Quantification of the percentage of cells expressing BLIMP1, OCT4, and both markers in aggregates cultured with Dox and stained on day 4.

(E) qRT-PCR of endogenous PGC-associated transcripts.

Data in (D) and (E) from three independent experiments, mean and SD shown. *p < 0.01, **p < 0.001. See also Figure S1.

sorted labeled cells were plated together with 5.8×10^3 parental cells per 3.8 cm^2 dish. Cells were exposed to definitive endoderm differentiation medium, then fixed and stained for SOX17 at day 4 (Figure S2D). The total number of mKO2-positive clones was determined, along with the number of SOX17-positive cells per clone and the clone

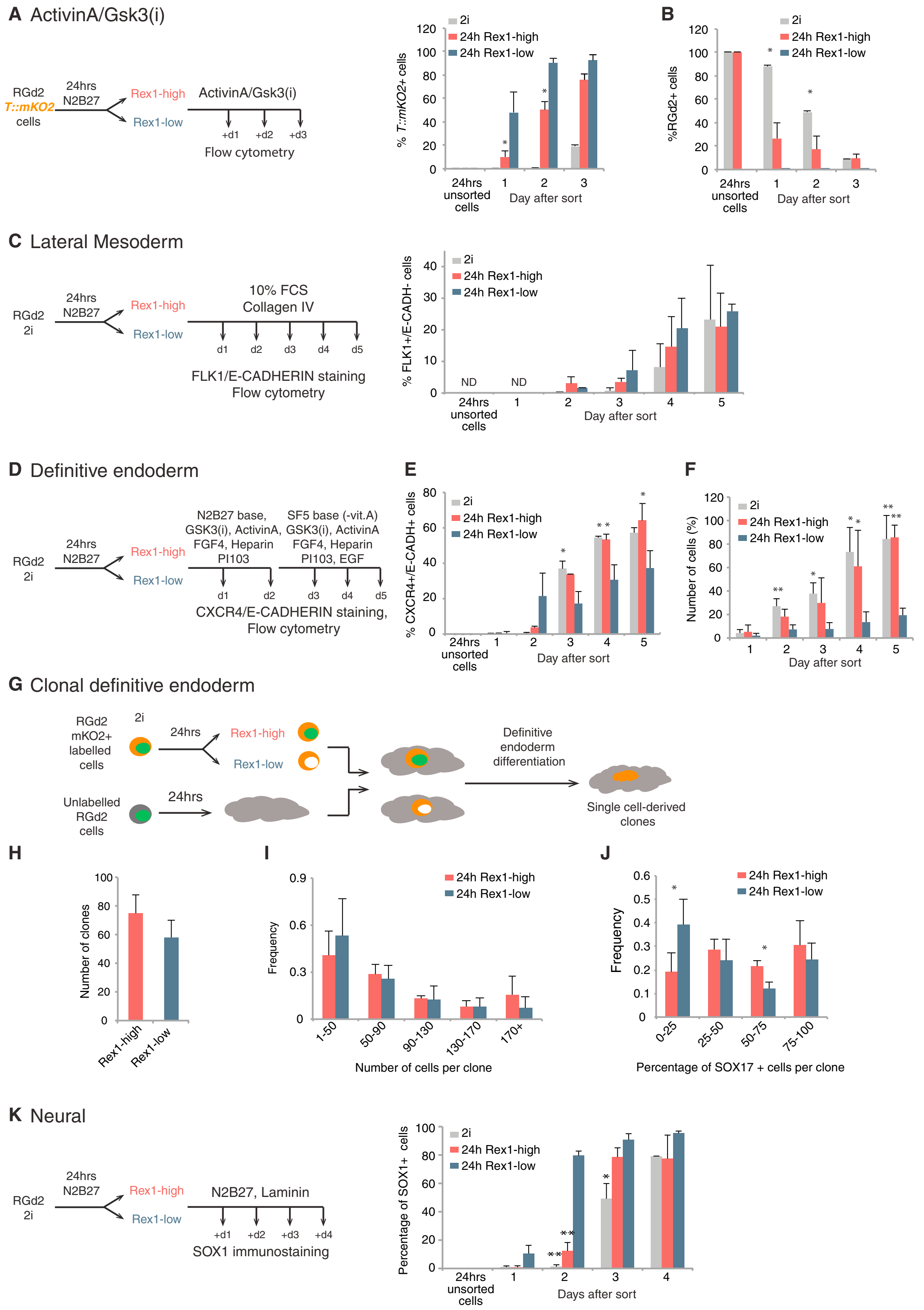

(legend on next page)

sizes. Similar numbers of clones and sizes were obtained from Rex1-high and -low cells (Figures 2H and 2I). The majority of Rex1-low cells were able to produce colonies containing SOX17-positive cells, although the output was less than from Rex1-high clones (Figure 2J).

Finally, we examined cell fate acquisition in N2B27 alone, which is permissive for neural differentiation (Ying et al., 2003). The great majority ($\geq$80%) of cells from both Rex1 fractions became immunopositive for SOX1, an exclusive marker of neuroectoderm (Pevny et al., 1998; Zhang et al., 2010) (Figure 2K). However, Rex1-low cells showed early upregulation of SOX1, with most cells becoming SOX1 positive on day 2, a day before the Rex1-high population (Figures 2K and S2E). In these conditions, cell viability and expansion were not significantly different between the populations (Figure S2F). Rex1-low cells subsequently also showed accelerated onset of expression of the neuronal marker type III **β**-TUBULIN (Lee et al., 1990) (Figure S2G).

Overall, these data indicate that after 24 hr of monolayer culture guided by autocrine cues, Rex1-low cells are competent for multi-lineage specification and respond more rapidly to induction than either ground-state ESCs or Rex1-high cells.

NODAL Does Not Regulate Kinetics of Exit from the Naive State

FGF4 is an autocrine factor that drives ESC transition via ERK signaling upon release from 2i (Betschinger et al., 2013; Kunath et al., 2007; Leeb et al., 2014; Stavridis et al., 2007). A second potential autocrine regulator is NODAL (Fiorenzano et al., 2016; Mullen et al., 2011; Ogawa et al., 2007). Detection of SMAD2 phosphorylation indicates that the pathway is active in ground-state ESCs, attributable to autocrine expression of NODAL (Figure S3A). Treatment with the ALK5/4/7 receptor inhibitor A83-01 (Alk(i)) (Tojo et al., 2005) eliminated SMAD2 phosphorylation after 30 min (Figure S3A). However, Alk(i) did not affect colony-forming capacity in 2i/LIF, even after continuous culture for three passages (Figure S3B), confirming that the NODAL pathway is not needed for maintenance of ground-state mouse ESCs.

We examined the contribution of autocrine NODAL pathway signaling in progression from the ESC state. We analyzed changes in gene expression in cells withdrawn from 2i in the continuous presence of Alk(i) (Figure 3A) and found no difference in the dynamics of downregulation of *Nanog* or *Klf2* mRNA (Figure 3B), nor of NANOG and KLF4 protein (Figures 3C and S3C). Functionally, the rate of decay in ESC clonogenicity was also unaffected (Figure 3D).

We evaluated expression of genes associated with the early post-implantation epiblast. Initial upregulation of *Fgf5* and *Otx2* was marginally reduced when NODAL signaling was inhibited (Figure 3E). However, these genes were subsequently downregulated more acutely on days 3 and 4 (Figure 3E). Conversely, transcripts for neuroectodermal lineage factors *Sox1*, *Zic1*, and *Pou3f3* were strongly upregulated in day-3/-4 Alk(i)-treated cultures, before appreciable expression in vehicle-treated cells (Figure 3F). At the protein level, most cells in Alk(i)-treated cultures had downregulated OCT4 and were SOX1 positive after 3 days, indicative of neural commitment, whereas control cultures at this time point displayed a mosaic pattern of co-exclusive SOX1 and OCT4 immunostaining (Lowell et al., 2006) (Figure 3G).

To validate findings with the inhibitor, we deployed small interfering RNAs (siRNAs) against NODAL signaling pathway components. In *Nodal*, *Smad2/3*, and *Tdgf1* knockdown experiments, the emergence of OCT4^{-}/SOX2^{+} and SOX2^{+}/SOX1^{+} cells was accelerated (Figures S3D and S3E). We conclude that suppression of NODAL

Figure 2. Multi-lineage Differentiation Capacity Is Manifest in Rex1-Low Cells

(A and B) Experimental set up and sample analysis for activin A + GSK3(i) treatment (A). Histogram shows the percentages of cells expressing *T:mKO2* or RGd2 (B).

(C) Experimental setup and sample analysis for lateral mesoderm differentiation. Histogram showing the percentage of FLK1^{+}/E-CAD^{-} cells.

(D) Experimental setup and sample analysis for definitive endoderm differentiation.

(E) Percentage of CXCR4^{+}/E-CADH^{+} double-positive cells.

(F) Normalized number of cells during definitive endoderm differentiation. The number of cells was normalized to the highest value obtained in that individual experiment.

(G) Single-cell analysis during definitive endoderm differentiation by seeding fluorescently labeled Rex1-Pos or Rex1-Neg cells at clonal density among unlabeled cells.

(H) Number of clones after 4 days of differentiation.

(I) Distribution of the number of cells per clone.

(J) Distribution of the percentage of SOX17-positive cells per clone.

(K) Experimental setup and sample analysis for neural differentiation. Histogram on the right shows the percentage of SOX1-positive cells during the differentiation time course.

Data from three independent experiments, mean and SD shown. *$p < 0.05$, **$p < 0.01$. See also Figure S2.

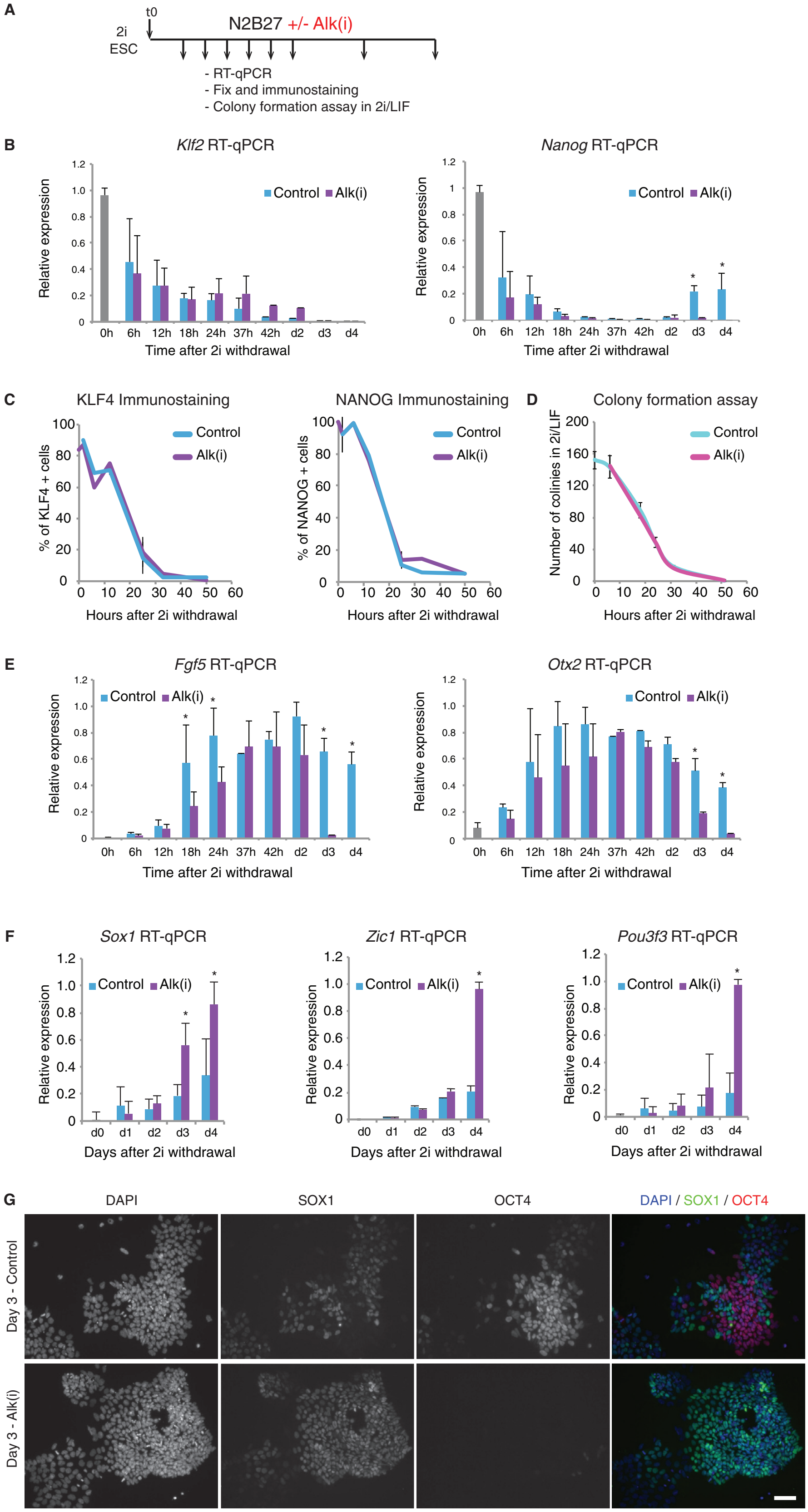

(legend on next page)

signaling does not substantially affect initial exit from the naive state but promotes subsequent specification to the neural lineage.

NODAL Signaling Potentiates Multi-lineage Differentiation

Interrogation of RNA-sequencing data from RGd2 sorted cells (Kalkan et al., 2017) revealed that NODAL pathway ligands, receptors, intracellular mediators, and target genes are expressed in undifferentiated ESCs and in 24-hr Rex1-high cells. Rex1-low cells, on the other hand, display reduced levels of *Nodal* and *Gdf3* transcripts and decreases in expression of the convertase *Pcsk6* (Pace4), as well as of pathway targets *Lefty1*, *Lefty2*, and *Smad6* (Figure S4A). This prompted treatment with Alk(i) only after sorting (Figure 4). We found that the Rex1-high population still responded by accelerated upregulation of SOX1 but the Rex1-low fraction showed no change, consistent with the pathway already being downregulated. These observations may explain why exogenous activin A is required to drive mesoendodermal lineage specification.

In light of these results, we postulated that NODAL signaling may function during the primary transition from naive pluripotency. We therefore inhibited the pathway for only the first 24 hr and analyzed the resulting Rex1-low cells. In line with results for continuous treatment, exposure to Alk(i) for 24 hr had little effect on downregulation of RGd2 (Figure S4B) or other naive pluripotency factor transcripts (Figure S4C). Upregulation of early post-implantation markers was also similar to that in vehicle-treated cells (Figure S4C). One day after sorting and replating, SOX1 protein was detectable only in a minority of untreated cells (Figure 5A). In contrast, up to half of cells generated after Alk(i) treatment upregulated SOX1 protein on day 1. Cell numbers appeared reduced at all time points for inhibitor-treated samples, although the difference was not statistically significant (Figure 5B).

We examined whether faster neural specification as a consequence of Alk(i) pre-treatment has consequences for other lineages. We analyzed the response of Alk(i)-treated cells to activin A/GSK3(i). Rex1-low cells showed a major reduction in the number of *T*::*mKO2*-positive cells (Figure 5C). Interestingly, this was mainly attributable to reduced total cell numbers after exposure to activin A/GSK3(i) (Figures 5D and S4D). A similar reduction in cell survival/proliferation was observed in cells exposed to lateral mesoderm differentiation conditions (Figures S4E–S4G). Thus, Rex1-low cells emerging after Alk(i) treatment appear to be compromised in their ability to respond to mesoderm-inducing signals. To evaluate endodermal specification, we employed the clonal mixing protocol described previously (Figure 5E). The total number of clones was not reduced (Figure S4H), but we observed a shift to smaller clones (Figure 5F) with fewer SOX17-positive cells (Figure 5G).

We assessed whether prior treatment with Alk(i) for 24 hr affected the potential of Rex1-low cells to respond to PGC-inducing transcription factors (Figure 5H). Alk(i)-treated cells produced less compact and smaller aggregates than control cultures (Figure 5H). The gene expression profile at days 2 and 4 of culture showed lower upregulation of endogenous *Prdm14*, *Nanos3*, and *Stella*, indicating significantly impaired PGCLC induction (Figure 5I). We similarly observed lower upregulation of endogenous *Nanos3*, *Tfap2c*, and *Stella* upon cytokine induction of PGCLCs (Figure S4I).

Collectively these findings indicate that suppression of NODAL signaling during exit from the ESC state reduces the capacity of cells to respond productively to inductive cues for mesoderm, endoderm, and germ cell specification.

Nodal Gene Deletion Compromises Germline and Somatic Lineage Specification

To confirm that the effect of Alk inhibitor treatment was indeed attributable to the absence of NODAL stimulation, we genetically inactivated *Nodal*. We employed CRISPR/Cas9 and used a pair of guide RNAs targeting the second and third exons (Figure S5A). Two clones negative for *Nodal* mRNA were identified and used for subsequent analyses (Figure S5B). Consistent with the inhibitor experiments,

Figure 3. Inhibition of Endogenous NODAL Signaling Does Not Affect Exit from the Naive State
(A) Experimental setup.
(B) Relative expression of pluripotency factors *Klf2* and *Nanog* over time when cells are differentiated in control (DMSO) or Alk(i).
(C) Percentage of KLF4- and NANOG-positive cells over time after 2i withdrawal when cells are differentiated in the presence or absence of Alk(i).
(D) Self-renewal capacity declines at a comparable rate for cells treated with vehicle control or Alk(i).
(E) Relative expression of post-implantation markers *Fgf5* and *Otx2* shows faster earlier downregulated for cells treated with Alk(i) over controls.
(F) Relative expression of neural-associated genes *Sox1*, *Zic1* and *Pou3f3* over time when cells are differentiated in controls or Alk(i).
(G) Inhibition of NODAL signaling results in accelerated reduction of OCT4 protein and increase in SOX1 protein at day 3 of differentiation. Scale bar, 50 μm.
Data from three independent experiments, mean and SD shown. *p < 0.01. See also Figure S3.

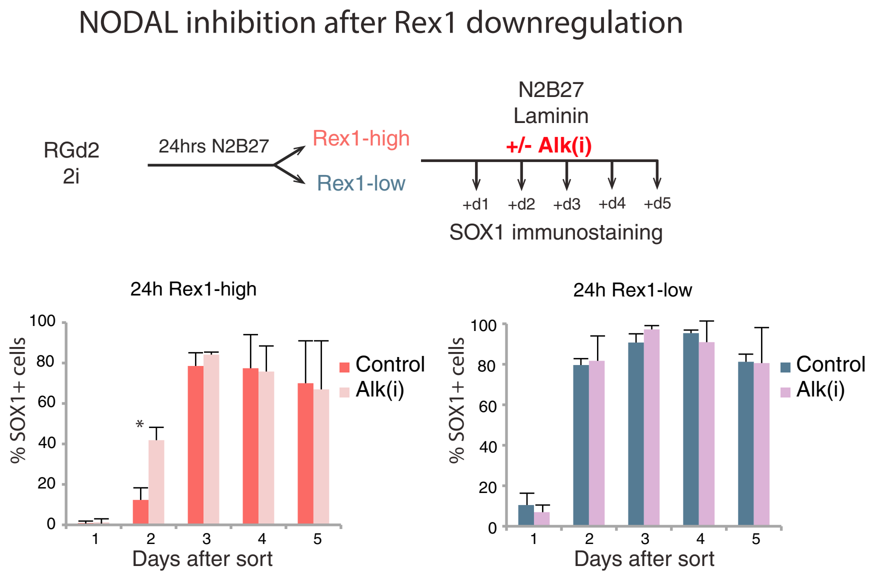

Figure 4. NODAL Signaling Acts during the Transition from the Naive State

Inhibition of Nodal signaling with Alki(i) in Rex1-high and Rex1-low sorted fractions. Graphs show percentages of SOX1-positive cells after sorting and replating in the presence or absence of Alk(i). Data from three independent experiments, mean and SD shown. *$p < 0.05$.

we observed no changes in the clonogenic capacity of *Nodal*-deficient ESCs in 2i/LIF (Figure 6A). The expression of key pluripotency or early post-implantation genes was also unaffected (Figure 6B). Expression of *Lefty1*, a NODAL target gene, was reduced to almost undetectable levels in the knockout clones but could be restored by addition of activin A to the culture medium (Figure 6B).

We examined the lineage propensity of 24-hr Rex1-low cells from *Nodal*-mutant cells. During cytokine induction of PGCLCs, mutant aggregates were less compact (Figure 6C) and the expression of PGC markers was significantly reduced. *Nodal* KO cells also displayed a marked reduction in generation of both SOX1- and BRACHYURY-positive cells in respective inductive culture conditions (Figures 6E and 6F). In either condition, fewer mutant cells survived compared with wild-type controls or activin A-treated mutant cells. The expression of *Gdf3* (Figure 6B), a *Vg-1* homolog that may elicit Nodal-like responses (Chen et al., 2006), might partly compensate for the absence of NODAL to enable residual lineage specification. Nonetheless, our results indicate that autocrine NODAL signaling during transition from naive pluripotency facilitates acquisition of multi-lineage competence.

DISCUSSION

The defined context of ground-state ESC culture provides opportunities for experimentally dissecting the interplay between intrinsic and extrinsic factors that mediate progression through pluripotency. Here we investigated the trajectory of ESCs released from the ground state with minimal extrinsic input. We isolated cells that have lost ESC identity within 24 hr based on downregulation of RGd2, corroborated functionally by extinction of self-renewal capability (Kalkan et al., 2017). These cells show gene expression features related to the peri-implantation epiblast (Kalkan et al., 2017). Capacitation for germline and somatic lineage specification may be acquired around this formative period (Hayashi et al., 2011; Smith, 2017). Indeed newly formed Rex1-low cells readily differentiated into the germline and somatic lineages. Furthermore, we found that endogenous NODAL signaling is crucial for robust multi-lineage competence of cells transitioning out of naive pluripotency.

Rex1-low cells show more rapid upregulation of lineage markers in response to inductive stimuli compared with ground-state ESCs or Rex1-high cells. They have also gained capacity for PGCLC induction. It has previously been established that responsiveness to germ cell induction cues or factors is not manifest in naive ESCs or the pre-implantation epiblast but is a property acquired during developmental progression (Hayashi et al., 2011; Nakaki et al., 2013). By transcription factor overexpression, very few BLIMP1/OCT4 double-positive cells could be obtained from the Rex1-high fraction, while the Rex1-low fraction generated them readily. Presumably, mis-expression of germ cell-determining transcription factors interferes with transition of undifferentiated ESCs or 24-hr Rex1-high cells to competence. For cytokine induction of PGCLCs, however, Rex1-high cells are evidently able to transition to a competent state. Slower upregulation of PGCLC markers in Rex1-high cells compared with Rex1-low cells is consistent with this explanation. Thus in the defined ESC system, capacity for PGCLC induction appears to be gained rather rapidly upon loss of Rex1.

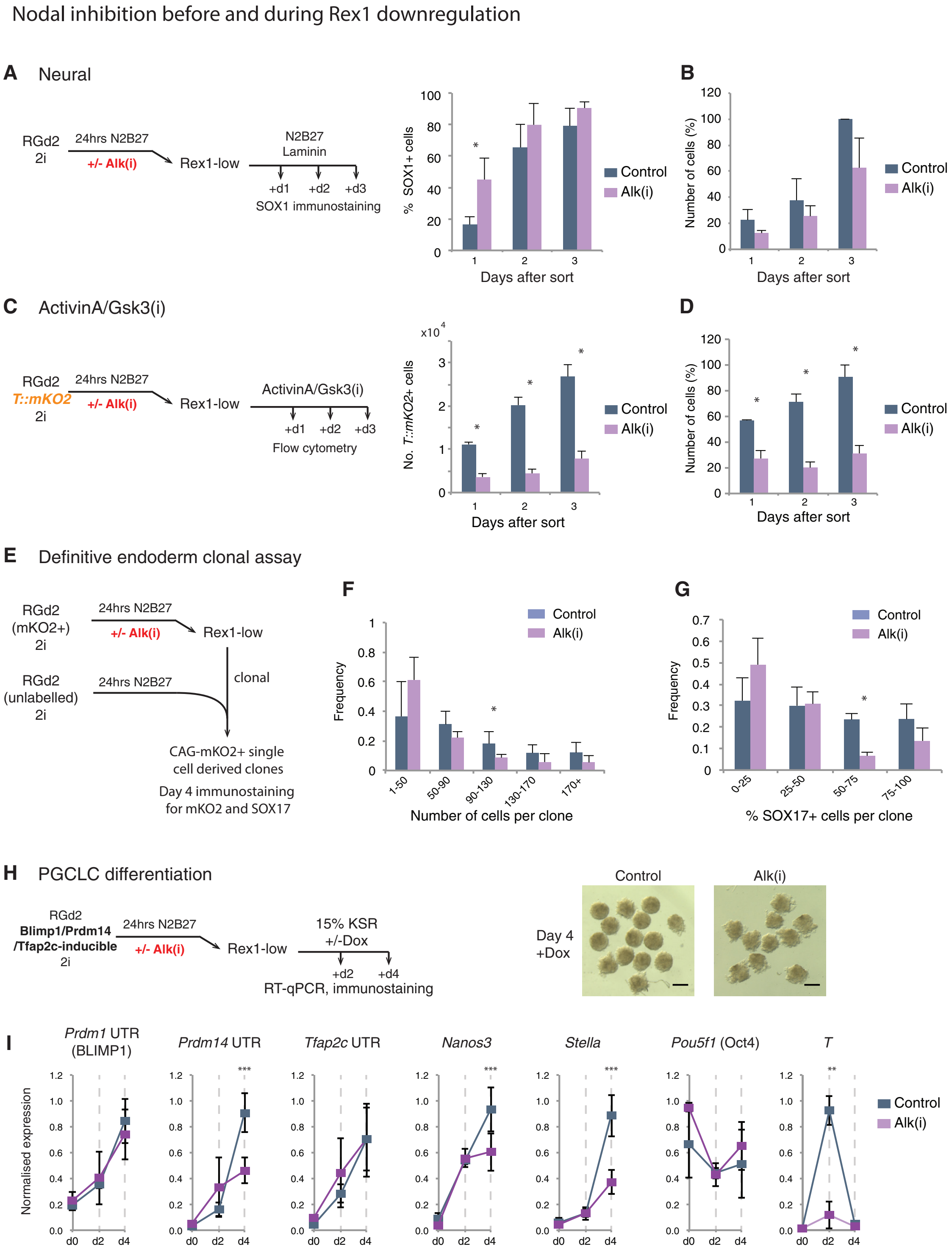

Figure 5. NODAL Signaling during Exit from the Naive State Prevents Precocious Neuralization

(A) Percentage of SOX1-positive cells arising from Rex1-low cells following control or Alk(i) treatment.

(B) Number of cells over the period analyzed in (A).

(C) Activin A/GSK3(i) induction of Alk(i) or control treated Rex1-low cells. Numbers of *T::mKO2*-positive cells, along with total cell numbers.

(legend continued on next page)

NODAL plays pleiotropic roles in the early embryo. Expression can be detected in the inner cell mass and persists throughout the epiblast until axis specification, when it becomes restricted to the proximal posterior region (Conlon et al., 1994; Mesnard et al., 2006). NODAL activity relies on pro-protein convertases, FURIN and PACE4, produced by the extra-embryonic ectoderm, which cleave and activate pro-NODAL (Beck et al., 2002; Mesnard et al., 2011). *Nodal*-deficient embryos die by embryonic day 7.5 (Conlon et al., 1994, 1991; Zhou et al., 1993). NODAL activity and autoinduction in the early post-implantation epiblast appears necessary to sustain pluripotency (Guzman-Ayala et al., 2004; Mesnard et al., 2006) and is dependent on paracrine provision of convertases by the extra-embryonic tissues (Beck et al., 2002). Mutant embryos show precocious upregulation of neural markers throughout the egg cylinder and fail to form a primitive streak (Brennan et al., 2001; Camus et al., 2006; Lu and Robertson, 2004). *Nodal* mutants also fail to specify the anterior visceral endoderm (Brennan et al., 2001), a signaling center essential for the establishment of anterior-posterior polarity. The multiple functions of NODAL, the complex interplay between extra-embryonic tissues and the epiblast, and the potential redundant activity of GDF3 (Chen et al., 2006) have complicated the precise delineation of its roles in pluripotency progression and lineage specification (Robertson, 2014).

Mouse ESCs express NODAL and exhibit phosphorylated SMAD2/3 (Mullen et al., 2011; Ogawa et al., 2004). Inhibition of NODAL signaling enhances SOX1 expression during differentiation (Matulka et al., 2013; Turner et al., 2014). Our results show that inhibition of NODAL signaling does not affect the acute downregulation of pluripotency factors when ground-state ESCs are released from 2i, in line with previous observations (Turner et al., 2014). Upregulation of early post-implantation markers is also unaffected. However, suppression of NODAL signaling compromises subsequent responses to inductive stimuli for mesoderm and endoderm, and results in precocious upregulation of neural markers. Interestingly, *Nodal* knockout ESCs exhibited a slightly different phenotype from inhibitor-treated cells. Mutant cells that exited the naive state showed reduced induction of both BRACHYURY and SOX1, with poor survival in both conditions. Lineage specification was not completely abolished, however, either because the requirement for NODAL pathway stimulation is not absolute, or possibly due to compensatory activity of GDF3. Both Alk(i)-treated and *Nodal*-mutant cells showed reduced induction of PCGLCs in response to either transcription factors or cytokines.

A key finding in this study is that the requirement for NODAL signaling is not restricted to the lineage priming stage but is apparent during initial transition from the ESC state, while cells are in the reversible Rex1-high condition (Kalkan et al., 2017; Martello and Smith, 2014). We have proposed that ESCs and naive epiblast cells transit through a formative phase of pluripotency during which they acquire competence for multi-lineage differentiation, including germline determination, prior to lineage priming (Kalkan and Smith, 2014; Smith, 2017). Formative cells are expected to respond to inductive signals rapidly and efficiently, as observed for Rex1-low cells at 24 hr. The molecular process of lineage capacitation remains unclear but is associated with reconfiguration of the transcription factor network, metabolic reprogramming, enhancer remodeling, and widespread epigenome and chromatin modification (Buecker et al., 2014; Choi et al., 2016; Dunn et al., 2014; Fiorenzano et al., 2016; Kalkan et al., 2017; Zylicz et al., 2015). Our findings point to a pivotal role for NODAL signaling in establishing formative pluripotency, in keeping with observations of a requirement for continuous NODAL activity to sustain pluripotency in the early post-implantation epiblast (Mesnard et al., 2006). Interestingly, SMAD2/3 is reported to be recruited by "master transcription factors" to regulatory loci in a cell type-specific manner (Mullen et al., 2011). In addition, a recent study in human ESCs suggested that SMAD2/3 is able to recruit histone methyltransferases to gene promoters (Bertero et al., 2015). Therefore, multi-lineage capacitation could depend upon the presence of SMAD2/3 at specific loci in ESCs during the transition from naive pluripotency.

EXPERIMENTAL PROCEDURES

Mouse ESC Culture and Differentiation

RGd2 ESCs were derived in 2i/LIF from heterozygous embryos (Kalkan et al., 2017). The RGd2/*T:mKO2* cell line was generated by targeting the endogenous *T* (BRACHYURY) locus with T2A-mKO2.

(D) To determine the normalized number of cells as a percentage for each independent experiment, we normalized the number of cells by the highest value obtained in that independent experiment.
(E) Experimental setup of definitive endoderm clonal assay.
(F) Distribution of the percentage of SOX17-positive cells per clone.
(G) Distribution of the number of cells per clone.
(H) Experimental setup of transcription factor-dependent PGCLC differentiation. Images show day-4 cultures in the presence of Dox from Alk(i)-treated and control cells. Scale bar, 1 mm.
(I) qRT-PCR assay of PGC-associated genes during induction process.
Data from three independent experiments, mean and SD shown. $^{*}p < 0.05$, $^{**}p < 0.01$, $^{***}p < 0.001$. See also Figure S4.

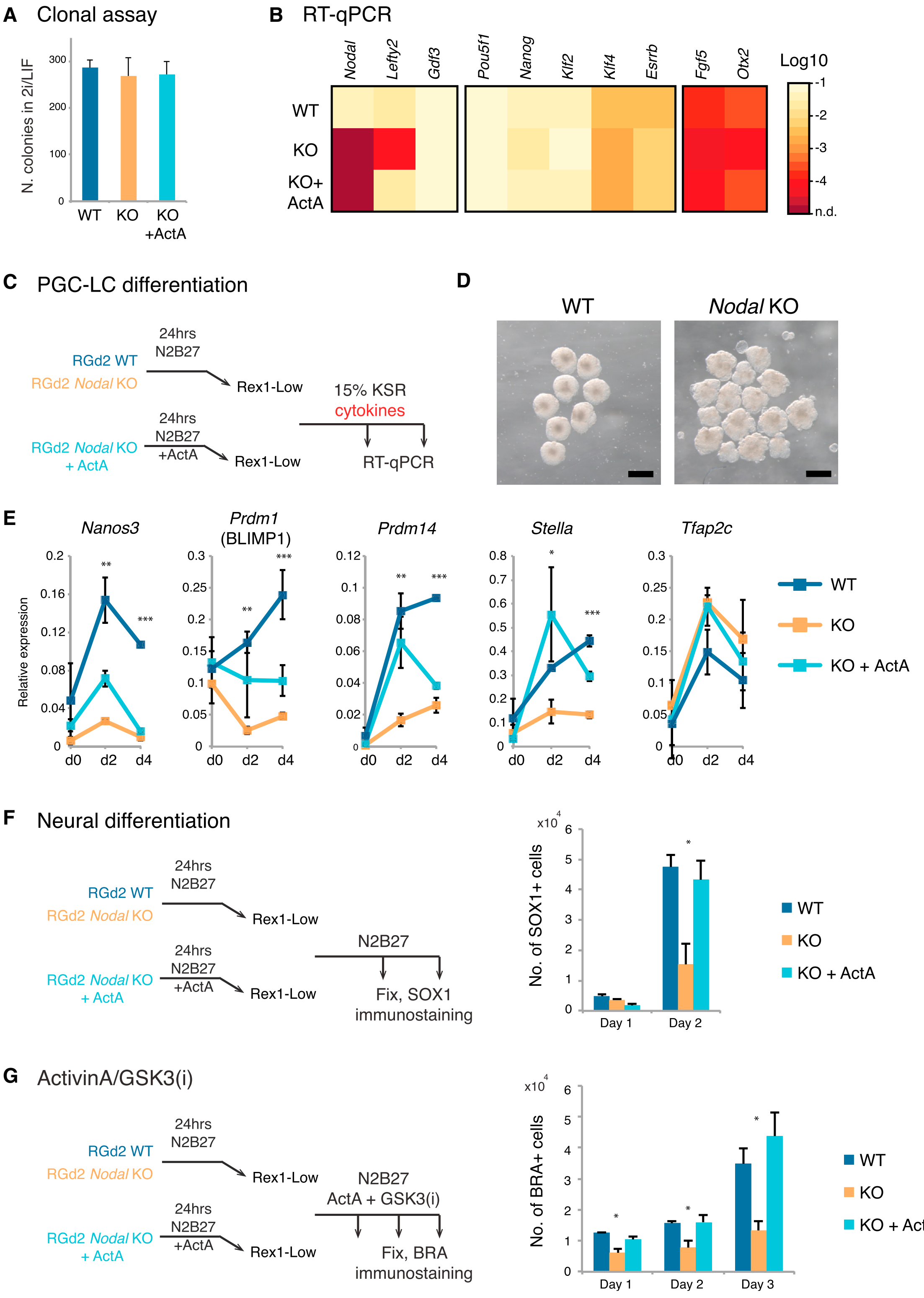

Figure 6. Deletion of *Nodal* Impairs Germline and Somatic Lineage Competence

(A) Colony-forming assay on wild-type (WT) and *Nodal*-mutant (KO) ESCs in 2i/LIF.

(B) Heatmap of marker expression determined by qRT-PCR in *Nodal* KO cells in 2i and in the presence of 5 ng/mL activin A. n.d., not detected.

(legend continued on next page)

ESCs were routinely maintained on gelatin-coated plates (Sigma, catalog no. 1890) in N2B27 medium (Stem Cells, SCS-SF-NB-02) supplemented with 1 μM PD0325901 and 3 μM Chir99021 (2i) without LIF, and passaged with Accutase (Millipore, SF006) every 2–3 days. For sorting experiments, cells were plated for 24 hr in 2i at 1.5×10^4 cells/cm^2 before washing once with PBS and changing the medium to N2B27. After 24–26 hr, cells were sorted by flow cytometry according to GFP levels into Rex1-high (highest 15%) and Rex1-low (lowest 15%) populations using a MoFlo sorter (Beckman Coulter). For neural differentiation, cells were plated at 1.0×10^4 cells/cm^2 on laminin-coated dishes (Sigma-Aldrich, L2020) in N2B27. Medium was changed every other day. For definitive endoderm induction (Morrison et al., 2015), 1.5×10^4 cells/cm^2 were plated in gelatin-coated plates directly in DE1, before switching to DE2 on day 2. DE2 was renewed on days 4 and 5. DE1 comprises batch-tested N2B27, supplemented with 3 μM Chir99021, 20 ng/mL activin A, 10 ng/mL recombinant mouse FGF4 (R&D Systems, 235-F4-025), 1 μg/mL heparin (Sigma-Aldrich, H3393), and 100 nM PI103 (Cayman Chemical, 10009209). DE2 medium is composed of SF5 base (DMEM/F12 [Life Technologies, 21331-020], 0.25% N2, 1% B27 without RA [Life Technologies, 12587-010], 0.05% BSA [Life Technologies, 15260-037], 0.1 mM β-mercaptoethanol [Life Technologies, 31350-010], 2 mM L-glutamine [Life Technologies, 25030-081]), supplemented with 3 μM Chir99021, 20 ng/mL activin A, 10 ng/mL FGF4, 1 μg/mL heparin, 100 nM PI103, and 20 ng/mL epidermal growth factor (EGF; Preprotech, AF-100-15). Lateral mesoderm differentiation (Nishikawa et al., 1998) was performed by plating 1.2×10^4 cells/cm^2 cells in collagen-coated plates (BD BioCoat, 354591) in Glasgow's minimum essential medium (GMEM; Sigma-Aldrich, G5154) with 10% batch-tested fetal calf serum (Sigma-Aldrich), 1× non-essential amino acids (NEAA; Life Technologies, 11140-050), 1 mM sodium pyruvate (Life Technologies, 11360-070), and 1 mM L-glutamine.

Activin A (20 ng/mL) and Chir99021 (3 μM) (GSK3(i)) treatment of sorted fractions was carried out on fibronectin-coated plates (Millipore, FC010) at 1.5×10^4 cells/cm^2. NODAL inhibitor experiments were carried out using A83-01 1 μM (Alk(i), Tocris Bioscience, 2939) with DMSO (1:10,000) as a carrier control.

Colony-forming assays were conducted by plating 100 cells/cm^2 per well in laminin-coated plates in 2i supplemented with 1,000 U/mL LIF (Wray et al., 2011). After 5 days, cells were stained using an alkaline phosphatase kit (Sigma, 86 R-1KT) and colonies counted.

For transcription factor induction of PGCLCs, the tri-cistronic Ap2g-T2A-Prdm14-P2A-Blimp1 fragment (APB1, a kind gift from Toshihiro Kobayashi and Azim Surani) was cloned into the phCMV*1-cHA-IRES-H2BBFP plasmid. pPyCAG-PBase, pPBCAG-rtTA-IN, and phCMV*1-APB-IRES-H2BBFP were co-transfected into RGd2 cells by TransIT-LT1 followed by G418 selection (400 μg/mL). For PGCLC induction, cells sorted at 24 hr for RGd2 expression were plated at 2,500 cells per well in a 96-well round-bottomed plate (Nakaki et al., 2013) in the presence or absence of 1 μg/mL doxycycline (Sigma-Aldrich) in GK15 medium (GMEM, 15% KSR [Sigma-Aldrich], 1× NEAA, 1 mM sodium pyruvate, 1 mM L-glutamine, 0.1 mM β-mercaptoethanol [Hayashi et al., 2011]). For cytokine induction of PGCLCs, 2,500 cells were plated in GK15 medium supplemented with 1,000 U/mL LIF, 500 ng/mL bone morphogenetic protein 2 (R&D), 100 ng/mL stem cell factor (R&D), and 50 ng/mL EGF. Aggregates were collected on days 2 and 4 for qRT-PCR.

To generate *Nodal* knockout clones, we transfected RGd2 cells with Cas9 and the guide RNAs CCT CTG CTC CTG AGG CCG GT and CAG TGG CTT GGT CTT CAC GG, which target exons 2 and 3, respectively. Single-cell-derived clones were picked after 55 hr of puromycin selection and a further 5 days of culture. Knockout clones were identified by qRT-PCR and cultured in parallel in either 2i or 2i supplemented with 5 ng/mL activin A (rescue). For differentiation studies with rescue cultures, activin A was present for the initial 24 hr until sorting.

Flow Cytometric Analysis of Fluorescent Reporters

Cells were dissociated into a single-cell suspension using Accutase and resuspended in PBS + 5% fetal bovine serum for analysis using an LSR Fortessa Analyzer (BD Biosciences).

Immunohistochemistry

Samples were fixed with 4% paraformaldehyde for 10 min at room temperature, permeabilized, and blocked for 2 hr with block buffer (PBS + 0.03% Triton X-100 + 3% donkey serum). Cells were incubated overnight at 4°C in block buffer with primary antibodies (Table S1). After three washes with PBS + 0.03% Triton X-100, cells were incubated with secondary antibodies (Life Technologies, 1:1,000) and DAPI in blocking buffer for 3 hr in the dark. After three washes with PBS + 0.03% Triton X-100, cells were left in PBS before imaging. Images were acquired using a Leica DMI3000 B inverted microscope and fluorescence in single cells quantified using CellProfiler (Jones et al., 2008). The number of cells was normalized for each independent experiment (e.g., highest density for a given independent experiment = 1).

Immunostaining of Surface Markers for Flow Cytometry

Cells were dissociated with enzyme-free Cell Dissociation Buffer (Life Technologies, 13151-014) at 37°C. Cells were resuspended with staining buffer (PBS + 1% rat serum) and incubated with directly conjugated antibodies (Table S1) for 30 min at 4°C in the

(C) Differentiation scheme for *Nodal* KO clones in inductive conditions for PGCLCs.
(D) Aggregate morphology on day 4 of PGCLC induction. Scale bar, 1 mm.
(E) qRT-PCR assay for PGC-associated genes during PGCLC induction.
(F) Experimental setup for neural differentiation. Number of SOX1-positive cells is shown.
(G) Activin A/GSK3(i) induction on WT and KO clones. Number of BRACHYURY-positive cells is shown.
Two independent *Nodal* KO clones from three independent experiments, mean and SD shown. *p < 0.01, **p < 0.001, ***p < 0.0001 in pairwise comparison of WT and KO. See also Figure S5.

dark. After three washes with staining buffer, cells were analyzed on an LSR Fortessa (BD Biosciences). Spherotech beads were used to quantify the number of cells. Undifferentiated ESCs stained with primary and secondary antibodies were used for FACS gating.

Gene Expression Analysis

RNA isolation from cell populations was performed with an RNAeasy kit (Qiagen). SuperScriptIII (Invitrogen) and oligo(dT) primers were used to synthesize cDNA. TaqMan, UPL, and SybrGreen probes were used (Table S2).

Gene Knockdown

Qiagen FlexiTube siRNAs for *Nodal*, *Tdgf1*, *Smad2*, and *Smad3* at a final concentration of 20 nM were used for gene knockdown. 1.5×10^4 cells/cm^2 were transfected in 24-well plates containing 500 μL of 2i medium with 0.5 μL of Lipofectamine RNAiMAX (Life Technologies, 13778075). After overnight incubation, cells were washed once with PBS before transfer to N2B27. Efficiency of transfection was quantified by flow cytometry on RGd2 cells transfected overnight with siRNA against GFP. Gene knockdown was quantified by qRT-PCR after overnight transfection.

Immunoblotting

Western blotting was performed using standard techniques. Primary antibodies (Table S1) were detected using peroxidase-conjugated secondary antibodies (Sigma-Aldrich, 1:5,000). Amersham ECL western blotting detection reagent (RPN2106) was used according to the manufacturer's instructions.

Statistics

ANOVA was used to compare three or more samples. Two-tailed Student's t test was used for pairwise comparisons. For all experiments, $n \geq 3$.

ACCESSION NUMBERS

The accession number for the RNA-sequencing data reported in Kalkan et al. (2017) is E-MTAB-5305.

SUPPLEMENTAL INFORMATION

Supplemental Information includes five figures and two tables and can be found with this article online at http://dx.doi.org/10.1016/j.stemcr.2017.05.033.

AUTHOR CONTRIBUTIONS

C.M., T.K., and A.S. designed the experiments. C.M. performed the experiments, analyzed the data, and prepared the figures. A.S. supervised the study. C.M. and A.S. wrote the paper.

ACKNOWLEDGMENTS

We thank Andy Riddell and Nigel Miller for flow cytometry and FACS support and Peter Humphreys for imaging support. We also thank Martin Leeb for creating and validating the TmKO2 reporter and Masaki Kinoshita for targeting and validated the RGd2/APB1 cell line. The APB1 construct was a kind gift from Toshihiro Kobayashi and Azim Surani. We thank Jennifer Nichols, Kevin Chalut, Graziano Martello, and Harry Leitch for discussions and comments on the manuscript. This research was funded by the Wellcome Trust (091484/Z/10/Z). The Cambridge Stem Cell Institute receives core support from the Wellcome Trust and the Medical Research Council (G1100526). C.M. was funded by a BBSRC studentship (961424). A.S. is a Medical Research Council Professor.

Received: December 13, 2016
Revised: May 24, 2017
Accepted: May 25, 2017
Published: June 29, 2017

REFERENCES

Arnold, S.J., and Robertson, E.J. (2009). Making a commitment: cell lineage allocation and axis patterning in the early mouse embryo. Nat. Rev. Mol. Cell Biol. *10*, 91–103.

Beck, S., Le Good, J.A., Guzman, M., Haim, N.B., Roy, K., Beermann, F., and Constam, D.B. (2002). Extraembryonic proteases regulate Nodal signalling during gastrulation. Nat. Cell Biol. *4*, 981–985.

Bertero, A., Madrigal, P., Galli, A., Hubner, N.C., Moreno, I., Burks, D., Brown, S., Pedersen, R.A., Gaffney, D., Mendjan, S., et al. (2015). Activin/Nodal signaling and NANOG orchestrate human embryonic stem cell fate decisions by controlling the H3K4me3 chromatin mark. Genes Dev. *29*, 702–717.

Betschinger, J., Nichols, J., Dietmann, S., Corrin, P.D., Paddison, P.J., and Smith, A. (2013). Exit from pluripotency is gated by intracellular redistribution of the bHLH transcription factor Tfe3. Cell *153*, 335–347.

Boroviak, T., Loos, R., Bertone, P., Smith, A., and Nichols, J. (2014). The ability of inner-cell-mass cells to self-renew as embryonic stem cells is acquired following epiblast specification. Nat. Cell Biol. *16*, 516–528.

Boroviak, T., Loos, R., Lombard, P., Okahara, J., Behr, R., Sasaki, E., Nichols, J., Smith, A., and Bertone, P. (2015). Lineage-specific profiling delineates the emergence and progression of naive pluripotency in mammalian embryogenesis. Dev. Cell *35*, 366–382.

Brennan, J., Lu, C.C., Norris, D.P., Rodriguez, T.A., Beddington, R.S., and Robertson, E.J. (2001). Nodal signalling in the epiblast patterns the early mouse embryo. Nature *411*, 965–969.

Buecker, C., Srinivasan, R., Wu, Z., Calo, E., Acampora, D., Faial, T., Simeone, A., Tan, M., Swigut, T., and Wysocka, J. (2014). Reorganization of enhancer patterns in transition from naive to primed pluripotency. Stem Cell *14*, 838–853.

Burtscher, I., and Lickert, H. (2009). Foxa2 regulates polarity and epithelialization in the endoderm germ layer of the mouse embryo. Development *136*, 1029–1038.

Camus, A., Perea-Gomez, A., Moreau, A., and Collignon, J. (2006). Absence of Nodal signaling promotes precocious neural differentiation in the mouse embryo. Dev. Biol. *295*, 743–755.

Chen, C., Ware, S.M., Sato, A., Houston-Hawkins, D.E., Habas, R., Matzuk, M.M., Shen, M.M., and Brown, C.W. (2006). The Vg1-related protein Gdf3 acts in a Nodal signaling pathway in the pre-gastrulation mouse embryo. Development *133*, 319–329.

Choi, H.W., Joo, J.Y., Hong, Y.J., Kim, J.S., Song, H., Lee, J.W., Wu, G., Schöler, H.R., and Do, J.T. (2016). Distinct enhancer activity of

Oct4 in naive and primed mouse pluripotency. Stem Cell Reports *7*, 911–926.

Conlon, F.L., Barth, K.S., and Robertson, E.J. (1991). A novel retrovirally induced embryonic lethal mutation in the mouse: assessment of the developmental fate of embryonic stem cells homozygous for the 413.d proviral integration. Development *111*, 969–981.

Conlon, F.L., Lyons, K.M., Takaesu, N., Barth, K.S., Kispert, A., Herrmann, B., and Robertson, E.J. (1994). A primary requirement for nodal in the formation and maintenance of the primitive streak in the mouse. Development *120*, 1919–1928.

Dunn, S.J., Martello, G., Yordanov, B., Emmott, S., and Smith, A.G. (2014). Defining an essential transcription factor program for naïve pluripotency. Science *344*, 1156–1160.

Fiorenzano, A., Pascale, E., Aniello, C.D.A., Acampora, D., Bassalert, C., Russo, F., Andolfi, G., Biffoni, M., Francescangeli, F., Zeuner, A., et al. (2016). Cripto is essential to capture mouse epiblast stem cell and human embryonic stem cell pluripotency. Nat. Commun. *7*, 1–16.

Gadue, P., Huber, T.L., Paddison, P.J., and Keller, G.M. (2006). Wnt and TGF-beta signaling are required for the induction of an in vitro model of primitive streak formation using embryonic stem cells. Proc. Natl. Acad. Sci. USA *103*, 16806–16811.

Gardner, R.L. (1975). Analysis of determination and differentiation in the early mammalian embryo using intra- and interspecific chimeras. Symp. Soc. Dev. Biol., 207–236.

Guzman-Ayala, M., Ben-Haim, N., Beck, S., and Constam, D.B. (2004). Nodal protein processing and fibroblast growth factor 4 synergize to maintain a trophoblast stem cell microenvironment. Proc. Natl. Acad. Sci. USA *101*, 15656–15660.

Habibi, E., Brinkman, A.B., Arand, J., Kroeze, L.I., Kerstens, H.H.D., Matarese, F., Lepikhov, K., Gut, M., Brun-Heath, I., Hubner, N.C., et al. (2013). Whole-genome bisulfite sequencing of two distinct interconvertible DNA methylomes of mouse embryonic stem cells. Cell Stem Cell *13*, 360–369.

Hayashi, K., Ohta, H., Kurimoto, K., Aramaki, S., and Saitou, M. (2011). Reconstitution of the mouse germ cell specification pathway in culture by pluripotent stem cells. Cell *146*, 519–532.

Jones, T.R., Kang, I.H., Wheeler, D.B., Lindquist, R.A., Papallo, A., Sabatini, D.M., Golland, P., and Carpenter, A.E. (2008). CellProfiler Analyst: data exploration and analysis software for complex image-based screens. BMC Bioinformatics *9*, 482.

Kalkan, T., and Smith, A. (2014). Mapping the route from naive pluripotency to lineage specification. Philos. Trans. R. Soc. Lond. B Biol. Sci. *369*. http://dx.doi.org/10.1098/rstb.2013.0540.

Kalkan, T., Olova, N., Roode, M., Mulas, C., Lee, H.J., Nett, I., Marks, H., Walker, R., Stunnenberg, H.G., Lilley, K.S., et al. (2017). Tracking the embryonic stem cell transition from ground state pluripotency. Development *144*, 1221–1234.

Kunath, T., Saba-El-Leil, M.K., Almousailleakh, M., Wray, J., Meloche, S., and Smith, A. (2007). FGF stimulation of the Erk1/2 signalling cascade triggers transition of pluripotent embryonic stem cells from self-renewal to lineage commitment. Development *134*, 2895–2902.

Kurimoto, K., Yabuta, Y., Ohinata, Y., Shigeta, M., Yamanaka, K., and Saitou, M. (2008). Complex genome-wide transcription dynamics orchestrated by Blimp1 for the specification of the germ cell lineage in mice. Genes Dev. *22*, 1617–1635.

Lee, M.K., Tuttle, J.B., Rebhun, L.I., Cleveland, D.W., and Frankfurter, A. (1990). The expression and posttranslational modification of a neuron-specific beta-tubulin isotype during chick embryogenesis. Cell Motil. Cytoskeleton *17*, 118–132.

Leeb, M., Dietmann, S., Paramor, M., Niwa, H., and Smith, A. (2014). Genetic exploration of the exit from self-renewal using haploid embryonic stem cells. Cell Stem Cell *14*, 385–393.

Leitch, H.G., McEwen, K.R., Turp, A., Encheva, V., Carroll, T., Grabole, N., Mansfield, W., Nashun, B., Knezovich, J.G., Smith, A., et al. (2013). Naive pluripotency is associated with global DNA hypomethylation. Nat. Struct. Mol. Biol. *20*, 311–316.

Lowell, S., Benchoua, A., Heavey, B., and Smith, A.G. (2006). Notch promotes neural lineage entry by pluripotent embryonic stem cells. PLoS Biol. *4*, e121.

Lu, C.C., and Robertson, E.J. (2004). Multiple roles for Nodal in the epiblast of the mouse embryo in the establishment of anterior-posterior patterning. Dev. Biol. *273*, 149–159.

Magnúsdóttir, E., Gillich, A., Grabole, N., and Surani, M.A. (2012). Combinatorial control of cell fate and reprogramming in the mammalian germline. Curr. Opin. Genet. Dev. *22*, 466–474.

Magnúsdóttir, E., Dietmann, S., Murakami, K., Günesdogan, U., Tang, F., Bao, S., Diamanti, E., Lao, K., Göttgens, B., and Azim Surani, M. (2013). A tripartite transcription factor network regulates primordial germ cell specification in mice. Nat. Cell Biol. *15*, 905–915.

Marks, H., Kalkan, T., Menafra, R., Denissov, S., Jones, K., Hofemeister, H., Nichols, J., Kranz, A., Stewart, A.F., Smith, A., and Stunnenberg, H.G. (2012). The transcriptional and epigenomic foundations of ground state pluripotency. Cell *149*, 590–604.

Martello, G., and Smith, A. (2014). The nature of embryonic stem cells. Annu. Rev. Cell Dev. Biol. *30*, 647–675.

Matulka, K., Lin, H.H., Hříbková, H., Uwanogho, D., Dvořák, P., and Sun, Y.M. (2013). PTP1B is an effector of activin signaling and regulates neural specification of embryonic stem cells. Cell Stem Cell *13*, 706–719.

Mesnard, D., Guzman-Ayala, M., and Constam, D.B. (2006). Nodal specifies embryonic visceral endoderm and sustains pluripotent cells in the epiblast before overt axial patterning. Development *133*, 2497–2505.

Mesnard, D., Donnison, M., Fuerer, C., Pfeffer, P.L., and Constam, D.B. (2011). The microenvironment patterns the pluripotent mouse epiblast through paracrine Furin and Pace4 proteolytic activities. Genes Dev. *25*, 1871–1880.

Morrison, G.M., Oikonomopoulou, I., Migueles, R.P., Soneji, S., Livigni, A., Enver, T., and Brickman, J.M. (2008). Anterior definitive endoderm from ESCs reveals a role for FGF signaling. Cell Stem Cell *3*, 402–415.

Morrison, G., Scognamiglio, R., Trumpp, A., and Smith, A. (2015). Convergence of cMyc and -catenin on Tcf7l1 enables endoderm specification. EMBO J. *35*, 356–368.

Mullen, A.C., Orlando, D.A., Newman, J.J., Lovén, J., Kumar, R.M., Bilodeau, S., Reddy, J., Guenther, M.G., DeKoter, R.P., and Young, R.A. (2011). Master transcription factors determine cell-type-specific responses to TGF-β signaling. Cell *147*, 565–576.

Nakaki, F., Hayashi, K., Ohta, H., Kurimoto, K., Yabuta, Y., and Saitou, M. (2013). Induction of mouse germ-cell fate by transcription factors in vitro. Nature *501*, 222–226.

Nichols, J., and Smith, A. (2012). Pluripotency in the embryo and in culture. Cold Spring Harb. Perspect. Biol. *4*, a008128.

Nishikawa, S.I., Nishikawa, S., Hirashima, M., Matsuyoshi, N., and Kodama, H. (1998). Progressive lineage analysis by cell sorting and culture identifies FLK1+VE-cadherin+ cells at a diverging point of endothelial and hemopoietic lineages. Development *125*, 1747–1757.

Niwa, H., Yamamura, K., and Miyazaki, J. (1991). Efficient selection for high-expression transfectants with a novel eukaryotic vector. Gene *108*, 193–199.

Ogawa, K., Matsui, H., Ohtsuka, S., and Niwa, H. (2004). A novel mechanism for regulating clonal propagation of mouse ES cells. Genes Cells *9*, 471–477.

Ogawa, K., Saito, A., Matsui, H., Suzuki, H., Ohtsuka, S., Shimosato, D., Morishita, Y., Watabe, T., Niwa, H., and Miyazono, K. (2007). Activin-Nodal signaling is involved in propagation of mouse embryonic stem cells. J. Cell Sci. *120*, 55–65.

Pevny, L.H., Sockanathan, S., Placzek, M., and Lovell-Badge, R. (1998). A role for SOX1 in neural determination. Development *125*, 1967–1978.

Robertson, E.J. (2014). Dose-dependent Nodal/Smad signals pattern the early mouse embryo. Semin. Cell Dev. Biol. *32*, 73–79.

Rossant, J. (1975). Investigation of the determinative state of the mouse inner cell mass. Development *33*, 979–990.

Rossant, J., and Tam, P. (2009). Blastocyst lineage formation, early embryonic asymmetries and axis patterning in the mouse. Development *136*, 701–713.

Smith, A. (2017). Formative pluripotency: the executive phase in a developmental continuum. Development *144*, 365–373.

Stavridis, M.P., Lunn, J.S., Collins, B.J., and Storey, K.G. (2007). A discrete period of FGF-induced Erk1/2 signalling is required for vertebrate neural specification. Development *134*, 2889–2894.

Tojo, M., Hamashima, Y., Hanyu, A., Kajimoto, T., Saitoh, M., Miyazono, K., Node, M., and Imamura, T. (2005). The ALK-5 inhibitor A-83-01 inhibits Smad signaling and epithelial-to-mesenchymal transition by transforming growth factor-beta. Cancer Sci. *96*, 791–800.

Toyooka, Y., Shimosato, D., Murakami, K., Takahashi, K., and Niwa, H. (2008). Identification and characterization of subpopulations in undifferentiated ES cell culture. Development *135*, 909–918.

Tsakiridis, A., Huang, Y., Blin, G., Skylaki, S., Wymeersch, F., Osorno, R., Economou, C., Karagianni, E., Zhao, S., Lowell, S., and Wilson, V. (2014). Distinct Wnt-driven primitive streak-like populations reflect in vivo lineage precursors. Development *141*, 1209–1221.

Turner, D.A., Trott, J., Hayward, P., Rué, P., and Martinez Arias, A. (2014). An interplay between extracellular signalling and the dynamics of the exit from pluripotency drives cell fate decisions in mouse ES cells. Biol. Open *3*, 614–626.

Wray, J., Kalkan, T., and Smith, A. (2010). The ground state of pluripotency. Biochem. Soc. Trans. *38*, 1027–1032.

Wray, J., Kalkan, T., Gomez-Lopez, S., Eckardt, D., Cook, A., Kemler, R., and Smith, A. (2011). Inhibition of glycogen synthase kinase-3 alleviates Tcf3 repression of the pluripotency network and increases embryonic stem cell resistance to differentiation. Nat. Cell Biol. *13*, 838–845.

Yamashita, J., Itoh, H., Hirashima, M., Ogawa, M., Nishikawa, S., Yurugi, T., Naito, M., and Nakao, K. (2000). Flk1-positive cells derived from embryonic stem cells serve as vascular progenitors. Nature *408*, 92–96.

Yang, S.-H., Kalkan, T., Morrisroe, C., Smith, A., and Sharrocks, A.D. (2012). A genome-wide RNAi screen reveals MAP kinase phosphatases as key ERK pathway regulators during embryonic stem cell differentiation. PLoS Genet. *8*, e1003112.

Yasunaga, M., Tada, S., Torikai-Nishikawa, S., Nakano, Y., Okada, M., Jakt, L.M., Nishikawa, S., Chiba, T., Era, T., and Nishikawa, S.I. (2005). Induction and monitoring of definitive and visceral endoderm differentiation of mouse ES cells. Nat. Biotechnol. *23*, 1542–1550.

Ying, Q.L., Stavridis, M., Griffiths, D., Li, M., and Smith, A. (2003). Conversion of embryonic stem cells into neuroectodermal precursors in adherent monoculture. Nat. Biotechnol. *21*, 183–186.

Ying, Q., Wray, J., Nichols, J., Batlle-Morera, L., Doble, B., Woodgett, J., Cohen, P., and Smith, A. (2008). The ground state of embryonic stem cell self-renewal. Nature *453*, 519–523.

Zhang, K., Li, L., Huang, C., Shen, C., Tan, F., Xia, C., Liu, P., Rossant, J., and Jing, N. (2010). Distinct functions of BMP4 during different stages of mouse ES cell neural commitment. Development *137*, 2095–2105.

Zhou, X., Sasaki, H., Lowe, L., Hogan, B.L., and Kuehn, M.R. (1993). Nodal is a novel TGF-beta-like gene expressed in the mouse node during gastrulation. Nature *361*, 543–547.

Zylicz, J.J., Dietmann, S., Günesdogan, U., and Hackett, J.A. (2015). Chromatin dynamics and the role of G9a in gene regulation and enhancer silencing during early mouse development. Elife *4*, e09571.

Stem Cell Reports, Volume *9*

Supplemental Information

NODAL Secures Pluripotency upon Embryonic Stem Cell Progression from the Ground State

Carla Mulas, Tüzer Kalkan, and Austin Smith

Figure S1

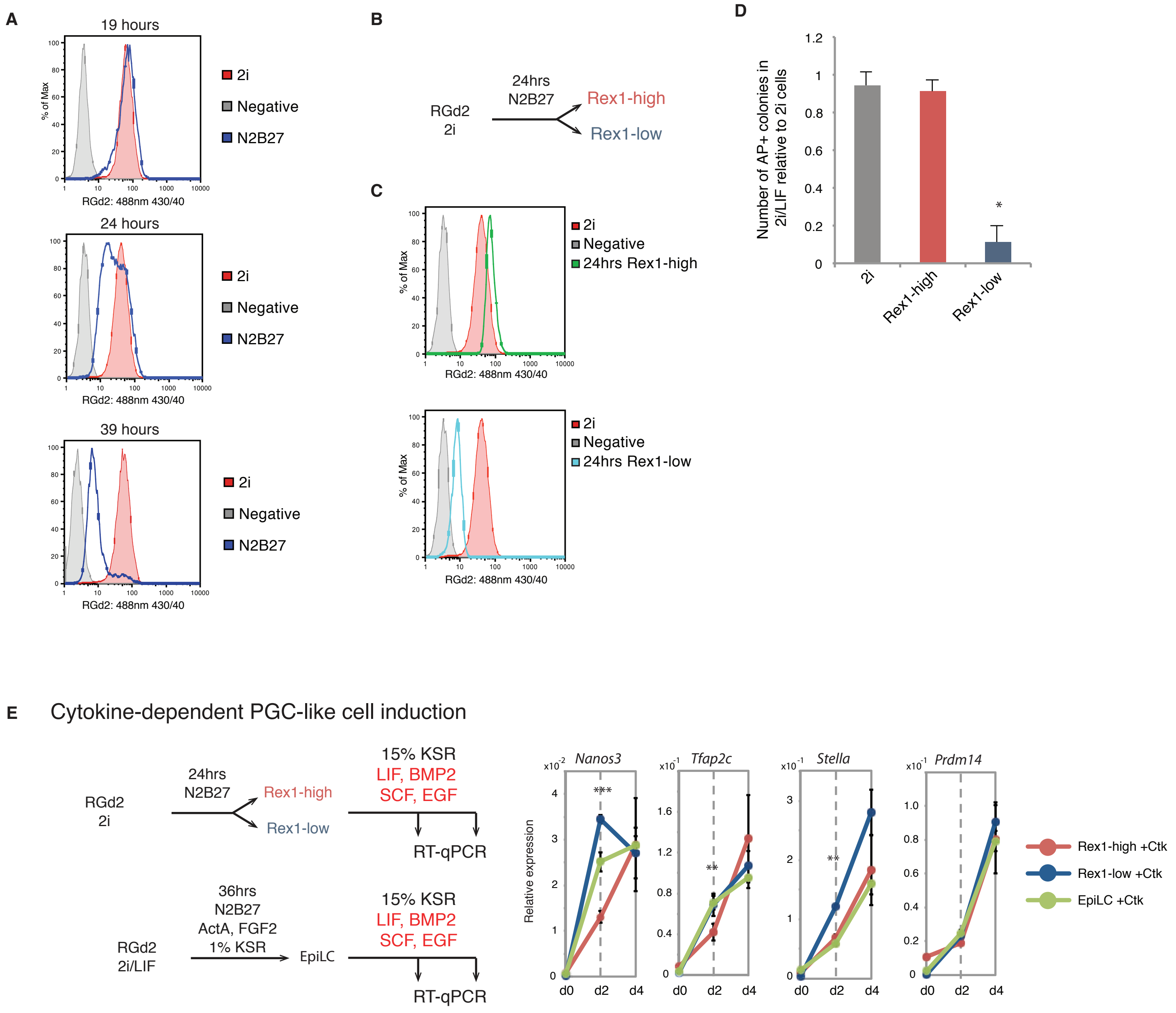

A
19 hours
24 hours
39 hours
% of Max
RGd2: 488nm 430/40
2i
Negative
N2B27
B
RGd2
2i
24hrs
N2B27
Rex1-high
Rex1-low
C
2i
Negative
24hrs Rex1-high
24hrs Rex1-low
D
Number of AP+ colonies in 2i/LIF relative to 2i cells
2i
Rex1-high
Rex1-low
E
Cytokine-dependent PGC-like cell induction
RGd2
2i
24hrs
N2B27
Rex1-high
Rex1-low
15% KSR
LIF, BMP2
SCF, EGF
RT-qPCR
RGd2
2i/LIF
36hrs
N2B27
ActA, FGF2
1% KSR
EpiLC
Nanos3
Tfap2c
Stella
Prdm14
Relative expression
d0
d2
d4
Rex1-high +Ctk
Rex1-low +Ctk
EpiLC +Ctk

Figure S2

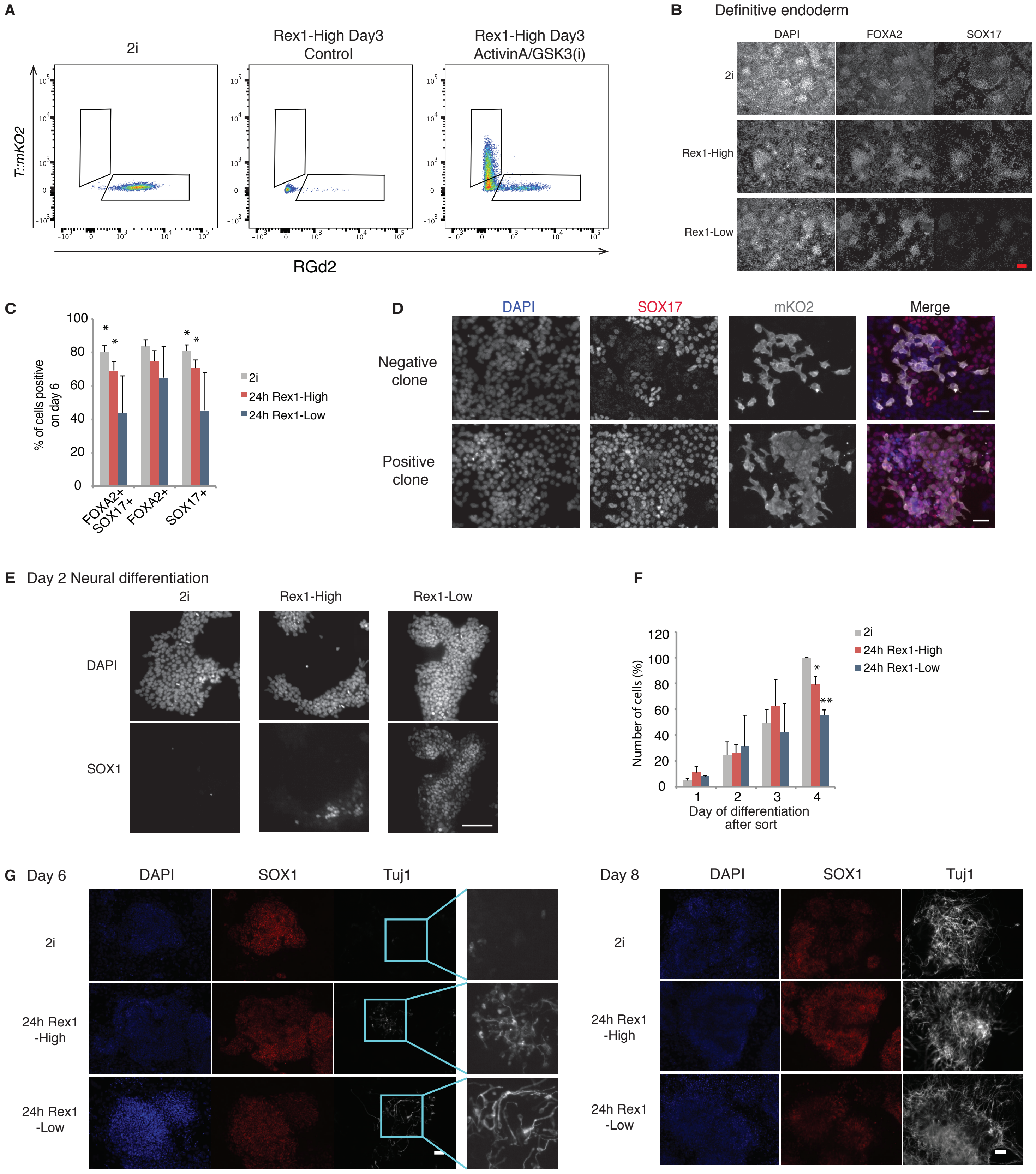

A
2i
Rex1-High Day3 Control
Rex1-High Day3 ActivinA/GSK3(i)
T::mKO2
RGd2
B
Definitive endoderm
DAPI
FOXA2
SOX17
2i
Rex1-High
Rex1-Low
C
% of cells positive on day 6
2i
24h Rex1-High
24h Rex1-Low
FOXA2+ SOX17+
FOXA2+
SOX17+
D
DAPI
SOX17
mKO2
Merge
Negative clone
Positive clone
E
Day 2 Neural differentiation
2i
Rex1-High
Rex1-Low
DAPI
SOX1
F
Number of cells (%)
Day of differentiation after sort
2i
24h Rex1-High
24h Rex1-Low
G
Day 6
DAPI
SOX1
Tuj1
2i
24h Rex1 -High
24h Rex1 -Low
Day 8

Figure S3

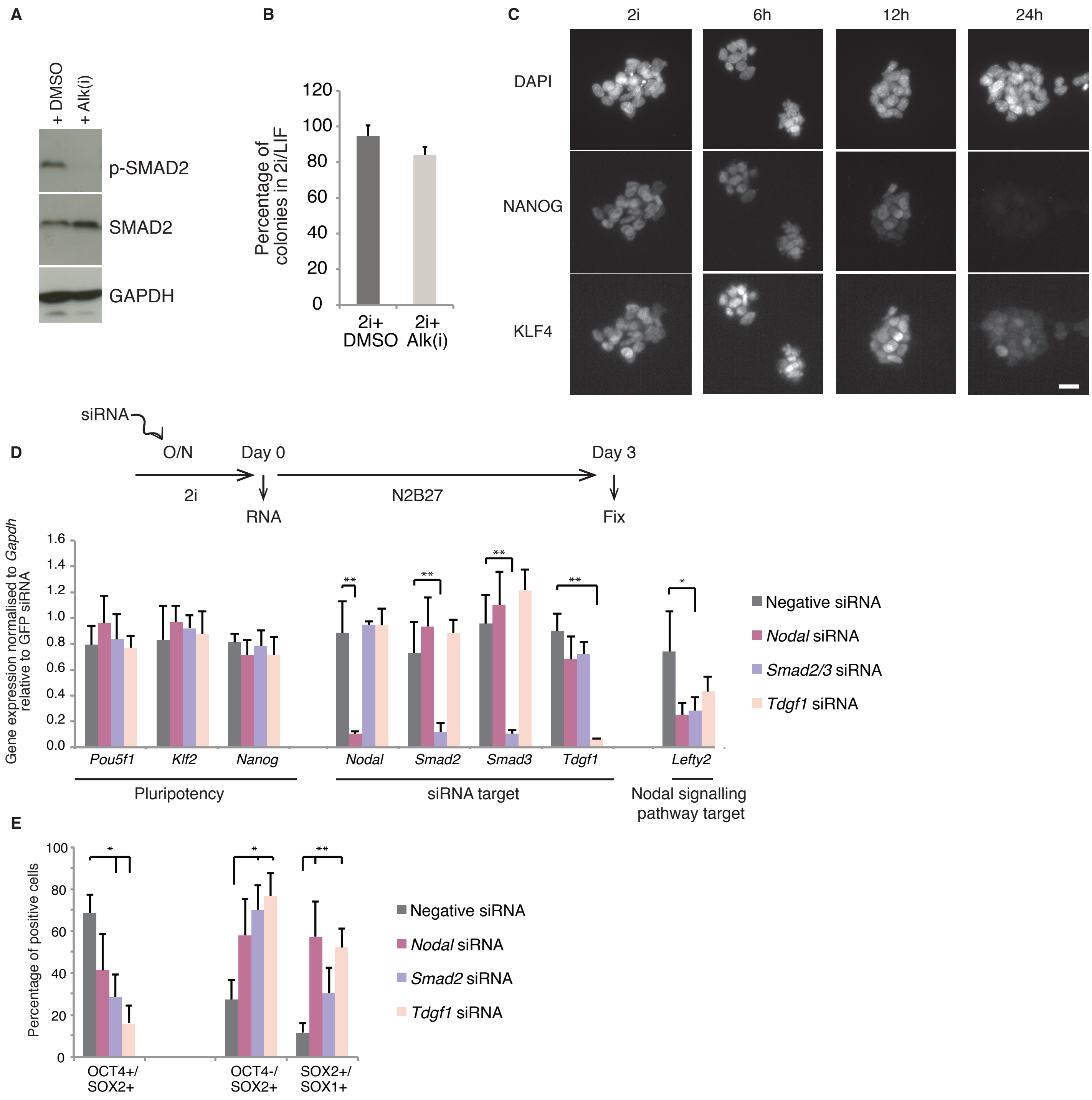
A
+ DMSO
+ Alk(i)
p-SMAD2
SMAD2
GAPDH
B
Percentage of colonies in 2i/LIF
120
100
80
60
40
20
0
2i+ DMSO
2i+ Alk(i)
C
2i
6h
12h
24h
DAPI
NANOG
KLF4
D
siRNA
O/N
Day 0
Day 3
2i
N2B27
RNA
Fix
Gene expression normalised to Gapdh relative to GFP siRNA
1.6
1.4
1.2
1.0
0.8
0.6
0.4
0.2
0.0
Pou5f1
Klf2
Nanog
Nodal
Smad2
Smad3
Tdgf1
Lefty2
Pluripotency
siRNA target
Nodal signalling pathway target
Negative siRNA
Nodal siRNA
Smad2/3 siRNA
Tdgf1 siRNA
E
Percentage of positive cells
100
80
60
40
20
0
OCT4+/ SOX2+
OCT4-/ SOX2+
SOX2+/ SOX1+
Negative siRNA
Nodal siRNA
Smad2 siRNA
Tdgf1 siRNA

Figure S4

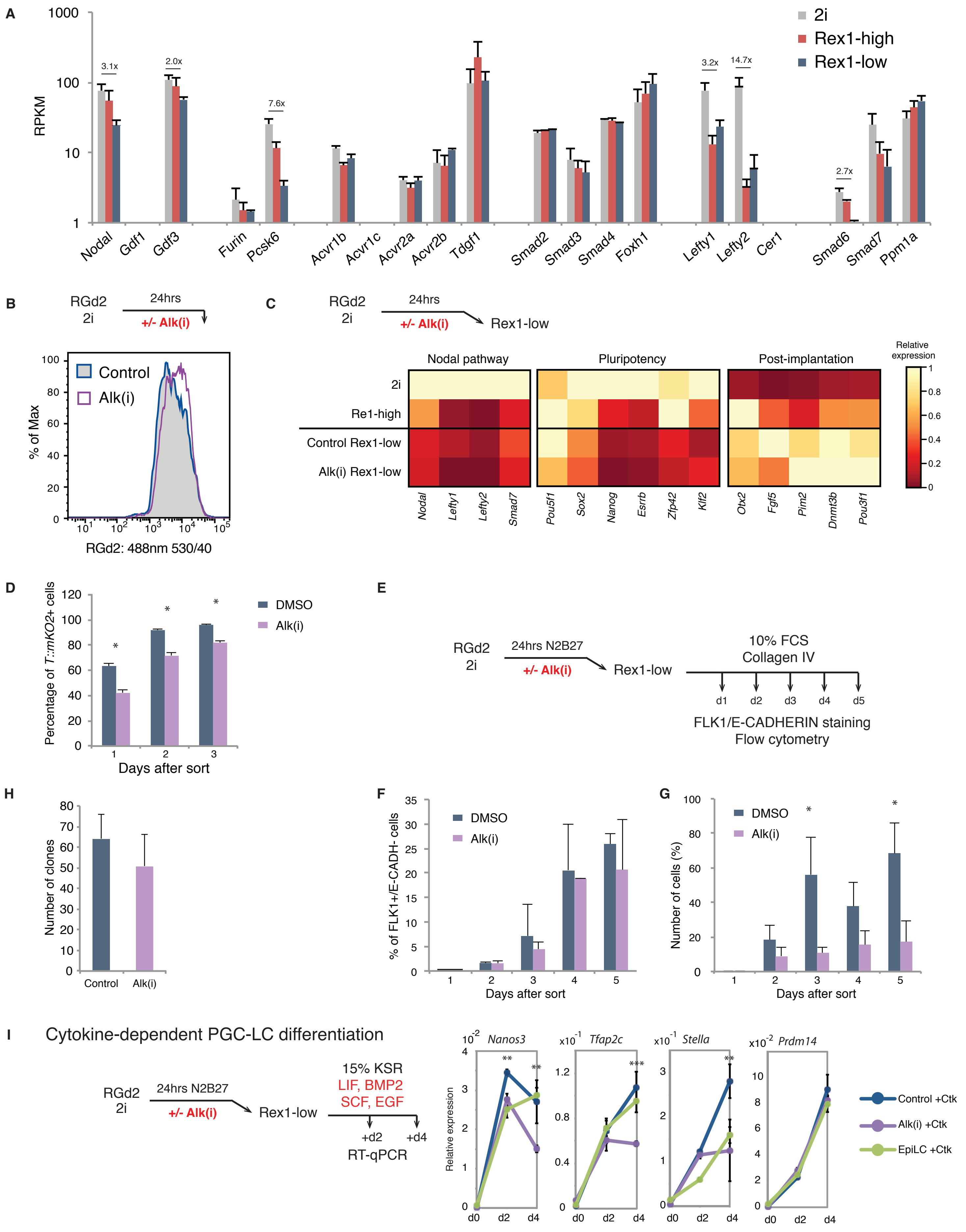
A
RPKM
2i
Rex1-high
Rex1-low
3.1x
2.0x
7.6x
3.2x
14.7x
2.7x
Nodal
Gdf1
Gdf3
Furin
Pcsk6
Acvr1b
Acvr1c
Acvr2a
Acvr2b
Tdgf1
Smad2
Smad3
Smad4
Foxh1
Lefty1
Lefty2
Cer1
Smad6
Smad7
Ppm1a
B
RGd2 2i
24hrs
+/- Alk(i)
Control
Alk(i)
% of Max
RGd2: 488nm 530/40
C
Rex1-low
Nodal pathway
Pluripotency
Post-implantation
Relative expression
2i
Re1-high
Control Rex1-low
Alk(i) Rex1-low
Nodal
Lefty1
Lefty2
Smad7
Pou5f1
Sox2
Nanog
Esrrb
Zfp42
Klf2
Otx2
Fgf5
Pim2
Dnmt3b
Pou3f1
D
Percentage of T::mKO2+ cells
DMSO
Alk(i)
Days after sort
E
24hrs N2B27
10% FCS
Collagen IV
d1
d2
d3
d4
d5
FLK1/E-CADHERIN staining
Flow cytometry
H
Number of clones
Control
Alk(i)
F
% of FLK1+/E-CADH- cells
G
Number of cells (%)
I
Cytokine-dependent PGC-LC differentiation
15% KSR
LIF, BMP2
SCF, EGF
+d2
+d4
RT-qPCR
Nanos3
Tfap2c
Stella
Prdm14
Relative expression
Control +Ctk
Alk(i) +Ctk
EpiLC +Ctk

Figure S5

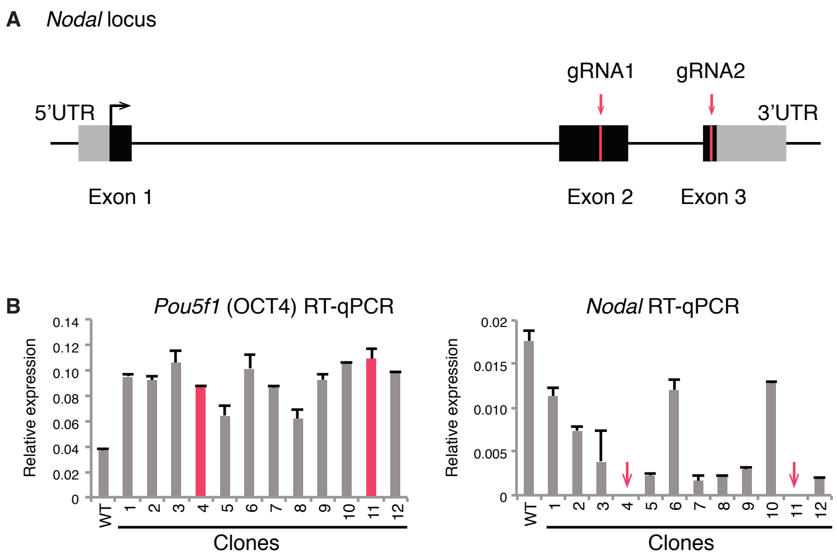
A
Nodal locus
gRNA1
gRNA2
5'UTR
3'UTR
Exon 1
Exon 2
Exon 3
B
Pou5f1 (OCT4) RT-qPCR
Relative expression
0.14
0.12
0.10
0.08
0.06
0.04
0.02
0
WT
1
2
3
4
5
6
7
8
9
10
11
12
Clones
Nodal RT-qPCR
Relative expression
0.02
0.015
0.010
0.005
0
WT
1
2
3
4
5
6
7
8
9
10
11
12
Clones

Table S1 – Antibodies used in this study

Antigen	Supplier	Cat. No	Dilution
SOX1	Cell Signalling	4194	1:200
OCT4	Santa Cruz	sc-5279 or sc-8628	1:400
NANOG	eBioscience	14-5761-80	1:200
KLF4	Abcam	ab72543	1:300
Tuj1	R&D	MAB1195	1:500
FOXA2	Abcam	ab40874	1:200
SOX17	R&D	AF1924	1:200
BRACHYURY	R&D	AF2085	1:200
ESRRB	Perseus	PP-H6705-00	1:300
mKO2	Amalgaam-MBL	M168-3	1:1000
BLIMP1	eBiosciences	14-5963-82	1:50
SMAD2	Cell Signalling	3101	1:1000
p-SMAD2	Cell Signalling	3103	1:1000
GAPDH	Sigma-Aldrich	G8795	1:5000
E-CADHERIN	eBioscience	50-3249-82	
CXCR4	BD Biosciences	552967 or 558644	
FLK1	BD Biosciences	562941	

Table S2 – Primers and probes used in this study

Target	Forward primer	Reverse primer	UPL probe
Actb	ggagattactgctctggctcc	acagagtacttgcgctcaggagg	-
Gdf3	tgttcgtgggaacctgct	ccatcttggaaaggtttctgtg	-
Lefty2	cacaagttggtccgtttcg	ggtacctcggggtcacaat	78
Nanos3	caaggcaaagacacaggatg	cttcctgccacttttggaac	25
Nodal	ccaaccatgcctacatcca	cacagcacgtggaaggaac	40
Pou3f1	catttttcgtttcgttttaccc	gagcgcagaccctctctg	72
Pou3f3	tctgagaccgcccacaag	gagcggcagtcagcaaag	22
Prdm1 UTR	ggtgaatcagggtgccttta	gagaggtgcagggaagcac	109
Prdm14 UTR	aaaatgaccttgaattacaggattaag	cataccaaatctctaggtagtgcaaa	76
Smad2	taagaatgagttttgtgaagggc	agcaaggagtacttgttactgtctg	-
Smad3	tccgtatgagcttcgtcaaagg	tagctcaatccagcagggg	-
Stella	tggaaattagaacgtacatactccaa	gatgcacaacgatccagattt	73
Tdgf1	gtttgaatttggacccgttg	ggaaggcacaaactggaaag	93
Tfap2c UTR	aaaagaggaaggaaacaggaaag	gggtcccctgtttttaagga	40
Zic1	aacctcaagatccacaaaagga	cctcgaactcgcacttgaa	7

TaqMan probes

Pou5f1
Sox2
Nanog
Esrrb
Zfp42
Klf2
Otx2
Fgf5
Pim2
Sox1
Dnmt3b
Gapdh

SUPPLEMENTAL FIGURE LEGENDS

Figure S1 – related to Figure 1

(**A**) Flow cytometry histogram of RGd2 cells after removal of 2i.

(**B**) Experimental set up for sorting experiments.

(**C**) Flow cytometry profile of sorted fractions.

(**D**) Replating capacity of 2i, 24hrs Rex1-high and 24hrs Rex1-low cells in 2i/LIF media.

(**E**) Experimental set up for cytokine-dependent PGCLC induction and RT-qPCR of endogenous PGC-associated transcripts. *** p<0.0001, **p<0.001 in pairwise comparison of Rex1-high and Rex1-low.

Figure S2 – related to Figure 2

(**A**) Flow cytometry plots of RGd2+*T::mKO2* cells in 2i, and Rex1-high sorted cells for 3 days in control or ActivinA+GSK3(i).

(**B**) Representative images of definitive endoderm differentiation immunostained for FOXA2 and SOX17. Scale bar= 50μm.

(**C**) Quantification of the percentage of cells positive for SOX17 and FOXA2 during definitive endoderm differentiation.

(**D**) Representative images of clonal definitive endoderm differentiation, showing an example of a clone predominantly SOX17 negative and a SOX17 positive clone.

(**E**) Representative images of neural differentiation on day 2. Scale bar= 50μm.

(**F**) Normalised number of cells during neural differentiation.

(**G**) Immunostaining for SOX1 and Tuj1 of 2i, Rex1-high and Rex1-low cells after 6 and 8 days of differentiation. Scale bar= 50μm.

To determine the normalised number of cells as a percentage for each biological replicate, the number of cells was normalised by the highest value obtained in that biological replicate.

*p<0.01

Figure S3 – related to Figure 3

(**A**) Western blot showing p-SMAD2, SMAD2 and GADPH protein in cells treated with control or Alk(i) for 30min.

(**B**) Number of colonies of ES cells grown in 2i+DMSO or 2i+Alk(i) for three passages.

(**C**) Representative images of NANOG and KLF4 immunostaining during the first 24hrs of differentiation (intensities have been enhanced for visualisation purposes). Scale bar= 20 μm.

(**D**) RT-qPCR of *Nodal*, *Tdgf1* and *Smad2/3* siRNA treated cells after overnight transfection in 2i. siRNA knockdown did not affect the expression of pluripotency genes *Pou5f1, Klf4* or *Nanog* but in some cases it did affect the expression of the NODAL signalling target *Lefty2.*

(**E**) Quantification of the number of OCT4/SOX2 double positive cells, OCT4 negative/SOX2 positive and SOX2/SOX1 double positive cells on day 3 of neural differentiation after treatment with siRNA.

* p <0.05, ** p<0.01

Figure S4 – related to Figures 4 and 5

(**A**) Expression of NODAL pathway signalling components in 2i, Rex1-high and Rex1-low cells (Kalkan et al. 2017). Fold changes are shown for genes showing a significant difference between 2i and Rex1-low cells (p<0.05).

(**B**) NODAL inhibition before and during downregulation of Rex1 – Flow cytometry plot of RGd2 cells differentiated for 24hrs in Alk(i) or control (DMSO).

(**C**) Relative expression of NODAL target genes, pluripotency and differentiation factors in Rex1-low cells arising from control or Alk(i) conditions by RT-qPCR. 2i and Rex1-high cells are included as controls.

(**D**) Percentage of *T::mKO2* positive cells during ActivinA/GSK3(i) treatment of control or Alk(i) derived Rex1-low cells.

(**E**) Lateral mesoderm differentiation of 24hrs Alk(i) or control treated Rex1-low cells.

(**F**) Percentage of FLK1+/E-CADH- cells.

(**G**) Number of clones after 4 days of definitive endoderm differentiation.

To determine the normalised number of cells as a percentage for each biological replicate, the number of cells was normalised by the highest value obtained in that biological replicate.

(**H**) Histogram showing the normalised number of cells.

(**I**) Experimental set up for cytokine-dependent PGCLC induction and RT-qPCR of endogenous PGC-associated transcripts of control or Alk(i)-treated cells.

*p<0.05, **p<0.001, *** p<0.0001 in pairwise comparison of Rex1-high and Rex1-low.

Figure S5 – related to figure 6

(**A**) Strategy for inactivating *Nodal*

(**B**) RT-qPCR for *Pou5f1* (Oct4) and *Nodal* in picked clones. Clones 4 and 11 were negative for *Nodal* mRNA and were used for subsequent analysis
